# NCAPH, ubiquitinated by TRIM21, promotes cell proliferation by inhibiting autophagy of cervical cancer through AKT/mTOR dependent signaling

**DOI:** 10.1038/s41419-024-06932-y

**Published:** 2024-08-06

**Authors:** Shiqi Wang, Xiaowen Qiao, Yaqi Cui, Liang Liu, Tamara Cooper, Yingxin Hu, Jiaxiang Lin, Haiting Liu, Meng Wang, John Hayball, Xiao Wang

**Affiliations:** 1 Department of Pathology, School of Basic Medical Sciences and Qilu Hospital, Shandong University, Jinan, Shandong Province China; 2 Key Laboratory of Carcinogenesis and Translational Research (Ministry of Education), Department of Gastrointestinal Surgery IV, Peking University Cancer Hospital & Institute, Beijing, 100142 China; 3 Experimental Therapeutics Laboratory, School of Pharmacy and Medical Sciences, University of South Australia Cancer Research Institute, Adelaide, SA Australia

**Keywords:** Gynaecological cancer, Oncogenes

## Abstract

Autophagy is closely related to the occurrence and development of human malignancies; however, the detailed mechanisms underlying autophagy in cervical cancer require further investigation. Previously, we found that the ectopic expression of NCAPH, a regulatory subunit of condensed protein complexes, significantly enhanced the proliferation of tumor cells; however, the underlying mechanisms were unclear. Here, we revealed that NCAPH is a novel autophagy-associated protein in cervical cancer that promotes cell proliferation by inhibiting autophagosome formation and reducing autophagy, with no effect on the cell cycle, apoptosis, or aging. Tripartite motif-containing protein 21 (TRIM21) is well known to be involved in inflammation, autoimmunity and cancer, mainly via its E3 ubiquitin ligase activity. Mass spectrometry and immunoprecipitation assays showed that TRIM21 interacted with NCAPH and decreased the protein stability of NCAPH via ubiquitination at the K11 lysine residue. Structural domain mutation analysis revealed that TRIM21 combined with NCAPH through its PRY/SPRY and CC domains and accelerated the degradation of NCAPH through the RING domain. Furthermore, TRIM21 promoted autophagosome formation and reduced cell proliferation by inhibiting NCAPH expression and the downstream AKT/mTOR pathway in cervical cancer cells. Immunohistochemical staining revealed that the protein expression of TRIM21 was negatively correlated with that of NCAPH and positively correlated with that of beclin-1 in cervical cancer tissues. Therefore, we provide evidence for the role of the TRIM21-NCAPH axis in cervical cancer autophagy and proliferation and the involvement of the AKT/mTOR signaling pathway in this process. These results deepen our understanding of the carcinogenesis of cervical cancer, broaden the understanding of the molecular mechanisms of TRIM21 and NCAPH, and provide guidance for individualized treatment of cervical cancer in the future.

## Introduction

Cervical cancer is a common malignant tumor of the female reproductive system worldwide. In China, nearly 130,000 new cases of cervical cancer are diagnosed each year, posing a serious threat to female health [1–3]. Cervical cancer is closely associated with persistent infection with human papillomavirus (HPV) [4, 5]. Nevertheless, it is important to note that HPV alone cannot induce cervical cancer. Furthermore, there is currently a lack of specific targeted treatments available for patients with cervical cancer in the clinic [6, 7]. Therefore, a detailed understanding of the molecular mechanism involved in the initiation and progression of cervical cancer is highly important for guiding prevention and treatment strategies and screening for new diagnostic and therapeutic targets for cervical cancer.

Autophagy is a process in which the components of the cytoplasm are wrapped by double-membrane vesicles to form autophagosomes, which are then transferred to lysosomes for degradation. It plays an important role in maintaining cell metabolism, internal environmental stability, and genome integrity [8–10]. In 2012, autophagy was confirmed to play a key role in inhibiting tumor occurrence and development [11]. It can inhibit tumor formation by limiting inflammation, removing unfolded toxic proteins and damaged mitochondria that generate reactive oxygen species [10]. Autophagy was also found to participate in the carcinogenesis of cervical cancer. It suppresses the proliferation of cervical cancer cells and stem cells, and suppresses tumorigenicity in mice, mechanisms that are intricately linked to the modulation of the AKT/mTOR signaling pathway [12–14]. However, the molecular mechanisms involved in autophagy require more in-depth investigation in cervical cancer.

NCAPH (non-SMC condensin I complex subunit H), located on chromosome 2q11.2, encodes a member of the Barr gene family and is a regulatory subunit of the condensed protein complex. It plays an important role in the transformation of interphase chromatin into condensed chromosomes [15, 16]. Recent studies have shown that NCAPH is upregulated in various human malignancies, leading to enhanced proliferation, migration, and invasion in colon, liver, and breast cancer. Furthermore, NCAPH promotes the cell cycle process, inhibits cell apoptosis, regulates endothelial cell migration and adhesion, and reduces DNA damage [17–22]. Therefore, NCAPH is a novel multifunctional carcinogenic protein involved in carcinogenesis. However, the underlying mechanism is largely unknown and requires further elucidation.

Previously, we identified NCAPH as a new oncoprotein able to promote cervical carcinogenesis by increasing the proliferation of cervical cancer cells [23]. In the present study, using in vitro and in vivo studies, we confirmed that NCAPH promoted cell proliferation by interfering with autophagosome formation and inhibiting autophagy. Furthermore, we showed that the E3 ubiquitin ligase TRIM21 was the upstream regulatory protein of NCAPH. It can bind to NCAPH via its PRY/SPRY and CC domains and promote its degradation by modifying the ubiquitination of the K11 lysine residue mediated by the RING domain. Furthermore, TRIM21 inhibited cell proliferation by hindering NCAPH-mediated activation of the AKT/mTOR pathway and autophagosome formation in cervical cancer.

## Materials and methods

### Cell culture and transfection

Cervical cancer cell lines (HeLa and SiHa) were purchased from the American Type Culture Collection (Manassas, VA, USA), and 293T cells were maintained in our laboratory. Short tandem repeat (STR) profile analysis was used to authenticate cells and exclude mycoplasma contamination. SiHa cells were cultured in RPMI-1640 medium, and HeLa and 293T cells were cultured in Dulbecco’s modified Eagle medium at 37 °C (Gibco, Shanghai, China). All cultures were supplemented with 10% fetal bovine serum (Gibco BRL, Grand Island, NY, USA) and 1% penicillin‒streptomycin.

Cell transfection was performed as previously described [23]. The siRNAs or plasmids were transfected into cells using Endofectin™ MAX transfection reagents (GeneCopoeia, Guangzhou, China). The deletion mutants of TRIM21 domains [ΔRING (15-58AA), ΔB-box (91-128AA), ΔCC (130-255AA) and ΔPRY/SPRY (286-475AA)], HA-TRIM21 (the plasmid with the full length of TRIM21 ORFs), and Flag-NCAPH (the plasmid with the full length of NCAPH ORFs) were constructed and purchased from Biosune Biotechnology (Shanghai) Co., Ltd. The His-Ub, HA-UB K11, HA-UB K48, and HA-UB K63 ubiquitination plasmids were gifts from Prof. Lihui Han (Department of Immunology, Shandong University). The specific siRNA sequences used were purchased from GenePharma (Shanghai, China) and are listed in Table 1.

**Table 1:** The sequences of siRNAs used in this study.

Gene Name	Sequence (5′→3′)
TRIM21-307 forward	CCAAUCGACAGCUAGCCAATT
TRIM21-307 reverse	UUGGCUAGCUGUCGAUUGGTT
NCAPH-forward	GCCAGAGUUAGGUUGUGUATT
NCAPH-reverse	UACACAACCUAACUCUGGCTT
Negative control forward	UUCUCCGAACGUGUCACGUTT
Negative control reverse	ACGUGACACGUUCGGAGAATT

Lentiviruses containing pGV248-TRIM21 shRNA and pGV248 control vector (carrying GFP) were synthesized by Shanghai Genechem Co., Ltd. HeLa and SiHa cells were infected with the virus according to the manufacturers’ instructions in the presence of 1 μg/ml puromycin for 6 weeks.

### Drug treatment

After transfection for 24 h, the cells were treated with different drugs as follows: rapamycin (RAPA, mTOR inhibitor, 5 μM), bafilomycin A1 (a blocker of autophagy‒lysosome fusion, 10 nM), trimethyladenine (3-MA, an autophagosome formation inhibitor, 25 μM), cycloheximide (CHX, a protein synthesis inhibitor, 1 μM), MG132 (a proteasome inhibitor, 10 μM), and MK2206 (an AKT inhibitor, 0.5 μM).

### Cell cycle analysis

The cell cycle distribution was analyzed using a cell cycle assay kit (Bestbio, Nanjing, China, BB-4104). Briefly, cells were digested and centrifuged at 1000 rpm for 5 min. The cell precipitate was immobilized in 75% ethanol and incubated with RNaseA solution and propidium iodide (PI) solution separately before flow cytometry analysis. The results were analyzed using Modfit LT 5 software.

### Apoptosis assay

Cell apoptosis was analyzed using an apoptosis assay kit (Bestbio, Nanjing, China, BB-4101). Briefly, the cells were centrifuged, and the precipitate was suspended in Annexin V binding solution. After incubation with Annexin V-FITC and PI solutions at 4 °C, the cells were subjected to flow cytometry analysis. The results were analyzed using FlowJo v.10.8.1 software.

Programmed cell death was further assessed using a TUNEL kit (Beyotime, Hebei, China, C1062M). Briefly, cells were fixed with 4% paraformaldehyde and incubated with 0.2% Triton X-100. After washing with PBS, the cultures were separately exposed to TdT-labeled reaction buffer, PI or DAPI (in PBS) before observation under a fluorescence microscope.

### β-Galactosidase staining

Cell senescence was analyzed with a β-galactosidase staining kit (Beyotime, Hebei, China, C0602). Cells were cultured in 6-well plates and fixed with β-galactosidase staining fixative at room temperature for 15 min. After incubation with the staining solution at 37 °C overnight, the cells were observed under an ordinary optical microscope.

### EdU and colony formation assays

Cell proliferation was detected with a Cell-Light™ EdU DNA Cell Proliferation Kit (Ribobio, Guangzhou, China). As previously described [23], the cells were labeled with EdU, reacted with Apollo reaction cocktail, and stained with 100 µL of Hoechst 33342 before observation under a fluorescence microscope (Olympus, Japan). The percentage of EdU-positive cells was defined as the proliferation rate. Colony formation was performed as previously described [23]. In brief, cells were transfected for 48 h, seeded into 6-well plates at 500 cells per well, and cultured for 14 days. The colonies were stained with crystal violet and counted. Each assay was repeated three times.

### Western blotting analysis

Western blotting analysis was performed as previously described [23]. Briefly, cells were lysed with CelLytic^TM^ MT Cell Lysis Reagent (Sigma, St. Louis, MO, USA). The protein concentration was determined using a BCA reagent kit (Beyotime, Shanghai, China). Details of the antibodies used are listed in Table 2. GAPDH was used as an internal control. The protein bands were visualized using an enhanced chemiluminescence kit (Beyotime, Shanghai, China).

**Table 2:** Primary antibodies used in this study.

Antigen	Dilution	Molecular weight	Supplier
TRIM21	1:1000 (WB) 1:100 (IHC)	55KD	Proteintech, China
NCAPH	1:1000 (WB) 1:100 (IHC)	83KD	Proteintech, China
GAPDH	1:10000	36KD	Proteintech, China
Beclin-1	1:1000 (WB) 1:50 (IHC)	60KD	Proteintech, China
P62	1:1000	65KD	Proteintech, China
ATG-7	1:1000	78KD	Huabio, China
ATG-5	1:1000	55KD	Huabio, China
AKT	1:1000	60KD	CST, USA
P-AKT	1:1000	60KD	CST, USA
mTOR	1:1000	289KD	CST, USA
P-mTOR	1:1000	289KD	CST, USA
LC3B	1:1000	14/16KD	CST, USA
HA tag	1:1000	\	Proteintech, China
His tag	1:1000	\	Proteintech, China
Flag tag	1:1000	\	Proteintech, China

*WB* western blotting, *IHC* immunohistochemical staining.

### Mass spectrometry analysis

HeLa cells were cultured and lysed for total protein extraction. The protein was precipitated by agarose beads with IgG antibody or anti-NCAPH antibody and loaded onto SDS-PAGE gels for silver staining. Total protein without immunoprecipitation was used as the ‘input’ group (positive control). After electrophoresis, the regions with the most significant differences between the IgG and NCAPH groups were excised and subjected to mass spectrometry for analysis.

### Co-immunoprecipitation (Co-IP)

The cells were collected, lysed on ice and centrifuged at maximum speed for 30 min. The supernatant was incubated with 1 μg of antibody at 4 °C overnight. Ten microliters of protein A agarose beads were pretreated with lysis buffer solution, added to antibody-containing cell lysate, and incubated at 4 °C for 2–4 h. The agarose beads were centrifuged, washed with lysis buffer and subjected to SDS-PAGE for western blot analysis after boiling in SDS loading buffer for 5 min.

### Transmission electron microscopy

The cells were prefixed in 2.5% glutaraldehyde at 4 °C for 2 h, washed with 0.1 M PBS buffer, and then fixed in 1% osmium tetroxide at 4 °C for 1 h. After additional washing with ddH2O, the samples were dehydrated in graded ethanol solutions (30%, 50%, 70%, 90% and 100%, separately) and twice in 100% acetone. Then, the samples were infiltrated into 1:3, 1:1 and 3:1 mixtures of epoxy resin and acetone at RT for 1, 4, and 12 h, respectively. Subsequently, the dehydrated cells were polymerized in epoxy resin at 37 °C for 12 h, 45 °C for 12 h, and finally 60 °C for 48 h. Sections with a thickness of 60 nm were prepared and stained with uranyl acetate for 10 min and then lead citrate for 5 min. After air drying, images were acquired via transmission electron microscopy (JEM-1200EX, JEOL; Tokyo, Japan). Considering that the background level of autophagy is low in cervical cancer cells and that siTRIM21 could further reduce autophagy, cells were first treated with 5 µM rapamycin for 2–4 h to induce the formation of autophagosomes and then with 20 µM chloroquine for 12 h to reduce the consumption of autophagosomes. The experiment was repeated three times.

### Autophagic flux monitoring

The mRFP-GFP-LC3 lentivirus double fluorescence autophagy indicator system (Hanbio, HB-AP210 0001) was used to monitor autophagic flux. Cells were infected with mRFP-GFP-LC3 lentivirus for 24 h following the manufacturer’s instructions. A confocal microscope (Carl Zeiss, LSM780) was used to observe autophagic flux. Autophagosomes and autolysosomes were detected by confocal microscopy analysis. The yellow spots indicate autophagosomes, and the red spots indicate autolysosomes.

### Immunofluorescence staining

Cells were cultured on a cover slip, washed with PBS, and fixed with 4% paraformaldehyde for 15 min. The cells were then incubated with 0.5% Triton X-100 for 20 min, goat serum for 30 min, and primary antibody overnight. On the second day, the cells were incubated with secondary fluorescent antibody at 20–37 °C for 1 h and then with DAPI in the dark for 5 min before observation under a fluorescence microscope. The experiment was repeated three times.

### LysoTracker Red and acridine orange staining

Cells were cultured in 96-well plates and washed with PBS three times. Then, LysoTracker Red and acridine orange (AO) were added at final concentrations of 50–75 and 8.5–17 µg/mL, respectively, and the cells were incubated at 37 °C. The cells were washed with PBS three times, and then 100 μL of Hoechst 33342 (1 μg/mL) was added for staining. After 10 min, the cells were observed with a fluorescence microscope.

### Ubiquitination-related experiments

Cervical cancer cells were transfected with siTRIM21 and HA-UB and then treated with MG132 at 24 h after transfection. The total protein in the cells was collected for coimmunoprecipitation (co-IP) and western blotting detection to determine the ubiquitination of the NCAPH protein. The 293T cell line was transfected with NCAPH and TRIM21 overexpression plasmids, followed by transfection of UB plasmids with three separate mutations of the lysine residue. After 48 h of transfection, the cells were treated with MG132 for 12 h, lysed for protein collection, and subjected to coimmunoprecipitation. NCAPH protein ubiquitination was detected by western blotting to verify the location of the TRIM21-regulated ubiquitination site in NCAPH.

### Patient population and immunohistochemical staining

In this study, 222 patients with invasive cervical squamous cell carcinoma (ICSCC) were enrolled. The paraffin samples were obtained between March 2007 and March 2011 at the Department of Pathology at Qilu Hospital. Hematoxylin and eosin (HE)-stained sections were reviewed by two experienced pathologists. If the diagnosis is inconsistent, the case will be discarded. The diagnoses were made according to the World Health Organization Classification of Tumors. Patient information was obtained from the patient medical records room at Qilu Hospital. The study was approved by the Ethics Committee of School of Basic Medical Sciences of Shandong University. Informed consent was obtained from all individual participants included in this study.

Immunohistochemical staining was performed as previously described [23], and the primary antibodies used are listed in Table 2. The staining results were recorded as previously described [23] and evaluated by two pathologists in a single-blinded manner.

### Statistical analysis

GraphPad Prism v.8.0 software was used for statistical analysis. Comparisons between groups were conducted by one-way ANOVA or two-way *t* tests. Differences between categorical variables were analyzed using the chi-square test and Spearman’s correlation test. The data are expressed as the means ± SDs. *p* < 0.05 was considered to indicate statistical significance.

## Results

### Knocking down NCAPH expression significantly promoted autophagy but had no effect on the cell cycle, apoptosis or aging of cervical cancer cells

We investigated the effects of NCAPH on the cell cycle, apoptosis, and aging of cervical cancer cells. NCAPH silencing did not significantly affect the cell cycle distribution of the HeLa or SiHa cells (*p* > 0.05) (Supplementary Fig. S1A–D). Furthermore, NCAPH elimination had no dramatic influence on the percentage of apoptotic cells (Supplementary Fig. S1E–L) and the proportion of senescent cells (Supplementary Fig. S1M–P) (all *p* values > 0.05). These data indicated that NCAPH had no effect on the cell cycle, apoptosis, or aging of cervical cancer cells.

**Fig. 1:**
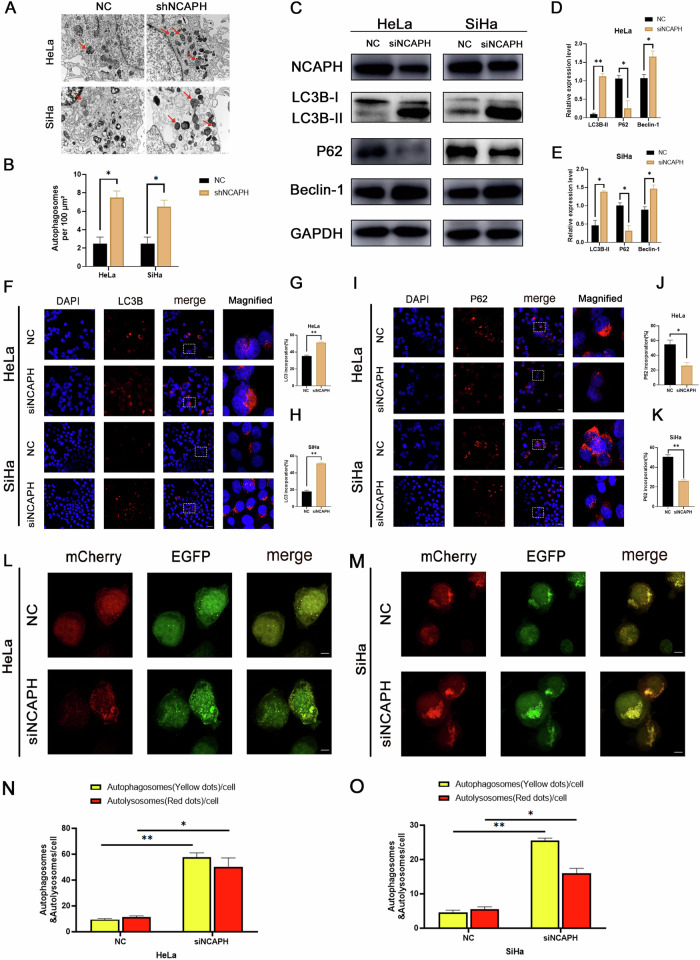
Interference with NCAPH promotes autophagy in cervical cancer cells. **A**, **B** The cervical cancer cell line with stable NCAPH knockdown was starved for 2-4 h in serum-free medium to induce autophagy and observed by transmission electron microscopy (TEM). The number of autophagosomes was calculated by quantitative analysis. Results revealed that interference with NCAPH significantly increased the number of autophagosomes in HeLa and SiHa cells (as indicated by the arrow). Data are representative images and means ± SD of 3 fields (100 μm^2^ per field). **C**–**E** Western blotting and statistical analysis revealed that a reduction in NCAPH expression increased the expression of LC3B II and Beclin-1 but significantly decreased that of P62. **F**–**K** Immunofluorescence staining revealed that interference with NCAPH increased LC3B expression but reduced that of P62 (scale = 50 µm). **L**, **M** After interference with NCAPH and treatment with bafilomycin A1, HeLa and SiHa cells were transfected with the lentivirus mCherry-EGFP-LC3 for 36–48 h. The yellow spots indicate autophagosomes, and the red spots indicate autolysosomes. The autophagic flow process was observed and analyzed by confocal microscopy. **N**, **O** ImageJ software was used to calculate the number of yellow and red puncta. The histogram shows the quantification of red and yellow spots. The figure shows the representative results of three experiments. *P < 0.05, **p < 0.01.

To confirm the relationship between NCAPH and autophagy, we subjected the cells to transmission electron microscopy (TEM). The number of autophagosomes was counted in 3 fields (100 μm^2^ per field), and data were shown as the Mean ± SDs. Results indicated that compared with the NC group (2.667 ± 0.333 per field for HeLa and SiHa), interference with NCAPH expression significantly increased the number of autophagosomes in HeLa (7.333 ± 0.333 per field), and SiHa cells (7.000 ± 0.577 per field) (*p* < 0.05) (Fig. 1A, B). Compared with those in the control group (NC), the expression levels of LC3 II and Beclin-1 were notably elevated, whereas those of P62 were diminished in cells transfected with siNCAPH (Fig. 1C–E). Consistently, immunofluorescence staining showed that after interfering with NCAPH, LC3 staining increased, while P62-labeled cells decreased significantly (Fig. 1F–K). Furthermore, interference with NCAPH significantly increased the spot ratio of mRFP to GFP, indicating that treatment promoted the autophagic process in cervical cancer cells (Fig. 1L–O). These results indicated that the downregulation of NCAPH led to a notable increase in autophagic activity in cervical cancer cells, suggesting that NCAPH plays a regulatory role in the autophagy process of cervical cancer cells.

### NCAPH regulated autophagy by inhibiting the synthesis of autophagosomes

**Fig. 2:**
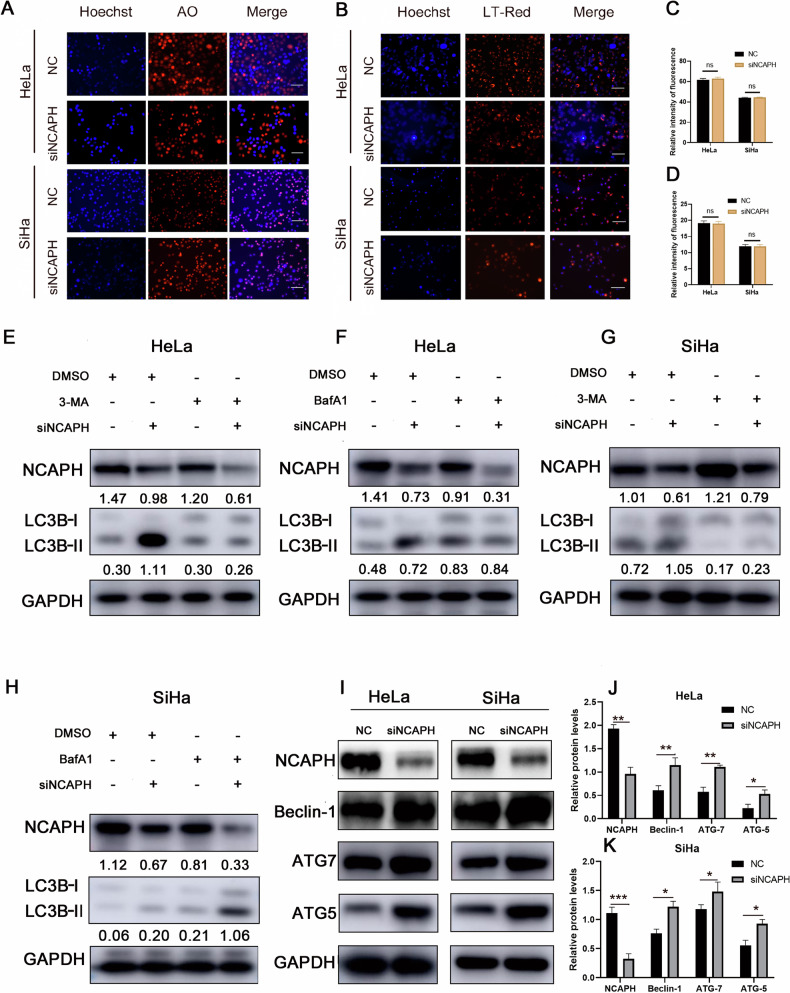
NCAPH inhibits autophagy by regulating the formation of autophagosomes. **A**–**D** Acridine orange and LysoTracker staining showed no changes in the red fluorescence signals after silencing NCAPH, indicating that NCAPH has no significant effect on the pH of lysosomes. **E**–**H** Western blotting analysis showed that 3-MA (2 mM) treatment almost completely eliminated the changes in LC3B II caused by NCAPH interference. In contrast, bafilomycin A1(100 nM) treatment did not reverse the increase in LC3B II expression caused by interference with NCAPH in cervical cancer cells; **I**–**K** Western blotting analysis showed that silencing NCAPH dramatically increased the protein expression of Beclin-1, ATG 5 and ATG 7. The figure shows the representative results of three experiments. *P < 0.05, **p < 0.01, ***p < 0.001.

To examine whether NCAPH could influence lysosomal activity, we evaluated lysosomal pH using LysoTracker Red and AO, which can stain acidic compartments, such as lysosomes. Elimination of NCAPH had no significant effect on the fluorescence of the cells (Fig. 2A–D). These findings suggested that the regulation of autophagy by NCAPH was not dependent on lysosomal pH and had no significant effect on the lysosomal fusion process. Western blotting analysis showed that interference with NCAPH could significantly increase the level of LC3 II in HeLa and SiHa cells, and 3-MA treatment eliminated the changes in the ratio of LC3 II caused by siNCAPH. Conversely, BafA1 treatment did not reverse the changes in the LC3 II ratio caused by siNCAPH (Fig. 2E–H). Thus, interference with NCAPH might affect the initial stages of autophagy and the formation of autophagosomes. To further verify this conclusion, we examined the effects of NCAPH on the expression of autophagic proteins associated with autophagosome formation (Fig. 2I–K). Consistent with these findings, the expression levels of beclin-1, ATG5, and ATG7 increased significantly after interference with NCAPH.

### The NCAPH protein was degraded by the ubiquitin proteasome pathway

**Fig. 3:**
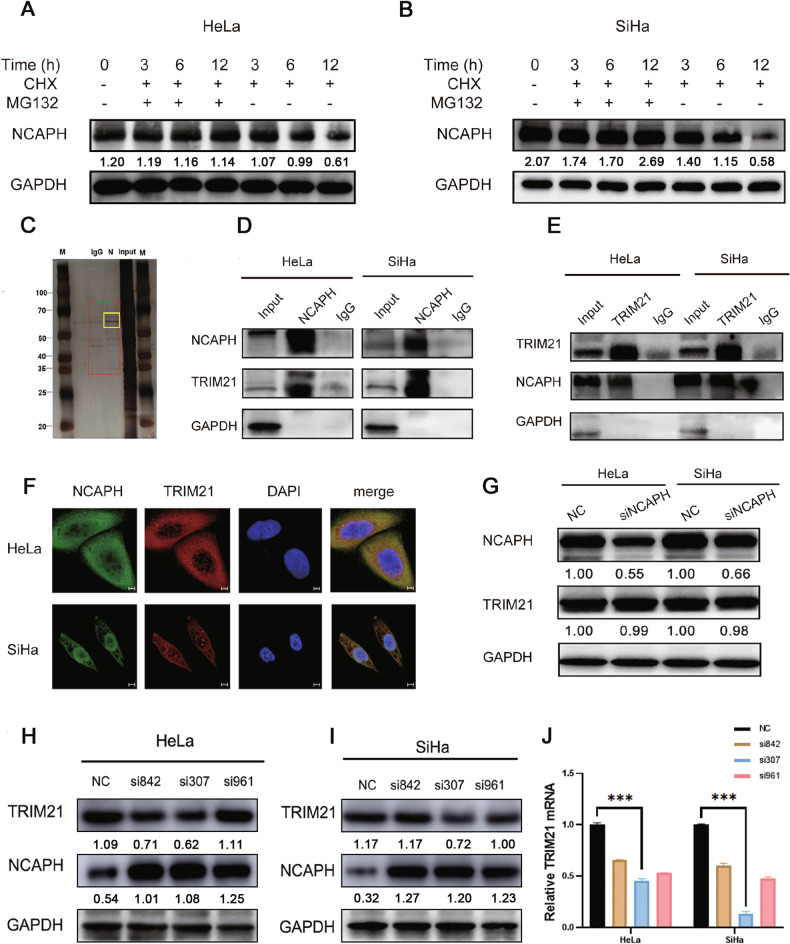
TRIM21 binds to NCAPH and regulates its protein expression. **A**, **B** HeLa and SiHa cells were treated with the protein synthesis inhibitor cycloheximide (CHX, 1 μM) and proteasome inhibitor MG132 (10 μM). Total cell proteins were collected for western blotting at different time points (0 h, 3 h, 6 h, and 12 h). The addition of MG132 slowed the degradation rate of the NCAPH protein, suggesting the involvement of the ubiquitin proteasome pathway. **C** Total protein in HeLa cells was immunoprecipitated by agarose beads containing IgG or NCAPH antibodies and silver stained after gel electrophoresis. The protein bands with the most significant differences were subjected to mass spectrometry analysis. **D**, **E** Coimmunoprecipitation was used to detect the interaction between TRIM21 and NCAPH. NCAPH and TRIM21 antibodies were used as separate baits; **F** Immunofluorescence assay showing the colocalization of the NCAPH and TRIM21 proteins in HeLa and SiHa cells. NCAPH, 488 nm, green fluorescence; TRIM21, 594 nm, red fluorescence; nucleus, DAPI, blue fluorescence. **G** Western blotting analysis showed that interference with NCAPH induced no changes in TRIM21 protein levels in cervical cancer cells. **H**–**J** Western blotting showed the efficiency of interference with three sets of siRNAs targeting TRIM21 and a significant increase in NCAPH protein levels after siTRIM21 treatment in HeLa and SiHa cells. ***P < 0.001.

To determine whether the NCAPH protein was regulated posttranslationally, we treated HeLa and SiHa cells with CHX and MG132. The results showed that when protein synthesis was inhibited, the protein levels of NCAPH gradually decreased over time (Fig. 3A, B). When proteasome function was inhibited by simultaneous MG132 treatment, the protein level of NCAPH decreased to that before treatment (Fig. 3A, B). These data suggested that the degradation of the NCAPH protein could be regulated by the ubiquitin proteasome pathway.

### The E3 ubiquitin ligase TRIM21 bound to the NCAPH protein through its ΔPRY/SPRY domain and CC domain

To elucidate the mechanism by which NCAPH is regulated at the posttranslational level, we compared protein bands extracted from HeLa cells treated with IgG or NCAPH antibodies and analyzed the 50–70 kDa regions with the most significant differences between the two groups by mass spectrometry. A total of 174 proteins were identified by the assay, of which eight had scores greater than 50: PKM2, TRIM21, CCT2, FH, HEL-S-123 m, GPI, EPHX1, and SHMT2. Considering the role of TRIM21 in the ubiquitin proteasome pathway, we targeted TRIM21 for further investigation (Fig. 3C). Coimmunoprecipitation experiments showed that TRIM21 could be precipitated by NCAPH, and vice versa (Fig. 3D, E). Immunofluorescence staining showed that the NCAPH protein was distributed mainly in the cytoplasm of HeLa cells, consistent with the pattern observed for TRIM21. Similarly, NCAPH was distributed in the cytoplasm and nucleus in SiHa cells, similar to TRIM21 (Fig. 3F). The data indicated the colocalization and interaction between the two proteins.

To determine whether NCAPH affects the protein level of TRIM21, we decreased the gene expression of NCAPH and found that it had no significant effect on the expression of TRIM21 (Fig. 3G). Furthermore, we designed three pairs of specific siRNAs targeting TRIM21, verified their interference efficiency in HeLa and SiHa cells, and selected si307 for subsequent experiments. When we interfered with TRIM21 expression, the protein level of NCAPH increased dramatically (Fig. 3H–J). The data confirmed that TRIM21 could reduce the protein level of NCAPH.

**Fig. 4:**
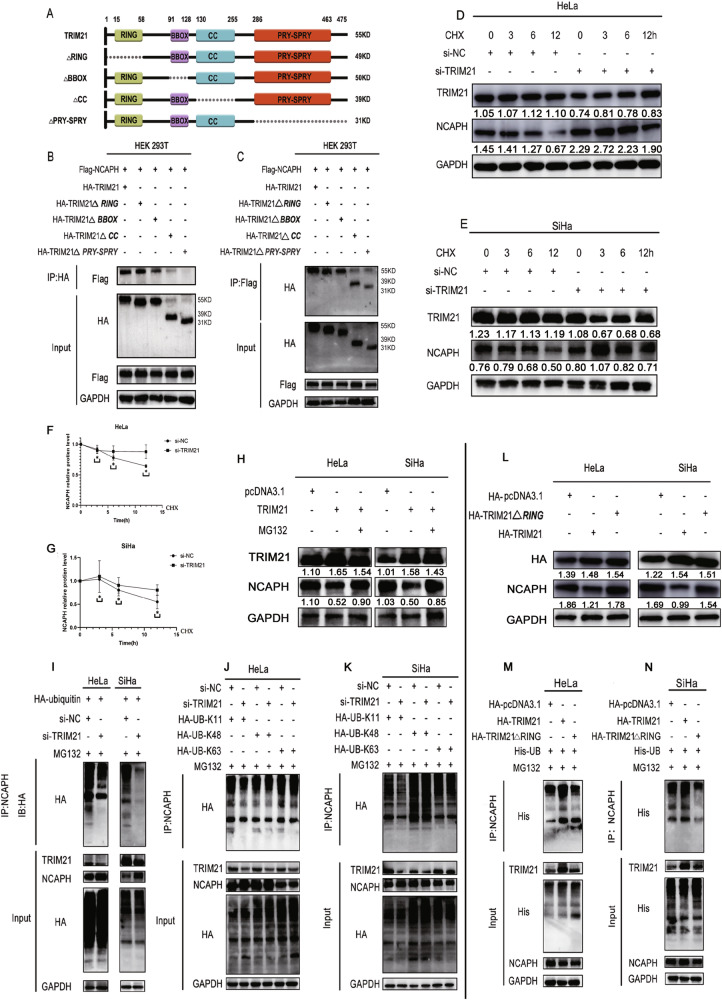
TRIM21 degraded the NCAPH protein via K11-mediated ubiquitination. **A** Construction of the TRIM21 truncation plasmid. Different colored boxes represent different structural domains, while discontinuous points represent deleted structural domains. **B**, **C** TRIM21 plasmids and corresponding domain mutants (labeled with HA) were cotransfected with NCAPH plasmids (labeled with Flag) into HEK 293T cells, and co-IP experiments were performed using Flag- or HA-labeled antibodies. Mutations in the PRY/SPRY and CC domains significantly influenced the binding between TRIM21 and NCAPH. **D**–**G** Cervical cancer cells were treated with cycloheximide (CHX), and total proteins were collected at different time points. Western blotting analysis revealed that, compared with the control group (siNC), knocking down TRIM21 significantly reduced the degradation rate of the NCAPH protein. **H** Western blotting analysis showed that MG132 treatment (10 μM) significantly reversed the decrease in NCAPH expression induced by TRIM21 overexpression. **I** HeLa and SiHa cells were transfected with siTRIM21 and HA-UB plasmids, respectively. Coimmunoprecipitation and western blotting assays showing a significant decrease in the ubiquitination level of NCAPH after interference with TRIM21. **J**, **K** HeLa and SiHa cells were transfected with siTRIM21 and cocultured with the HA-UB-K11, HA-UB-K48, or HA-UB-K63 plasmid. Coimmunoprecipitation and western blotting showing the effects of interfering with TRIM21: ubiquitination at the K11 position of NCAPH is significantly decreased. **L** Western blotting analysis showed that the mutation in the RING domain of TRIM21 resulted in the loss of regulation of NCAPH expression, suggesting that TRIM21 regulates NCAPH expression through its E3 ubiquitin ligase activity. **M**, **N** HeLa and SiHa cells were transfected with HA-TRIM21 or HA-TRIM21 Δ RING and cocultured with the His-UB plasmid. Coimmunoprecipitation and western blotting showed that TRIM21 overexpression significantly increased the ubiquitination level of NCAPH, while the elimination of the RING domain of TRIM21 significantly decreased it. The HA pcDNA3.1 vector was used as a control. *P < 0.05.

TRIM21 consists of three zinc binding domains, one RING domain, one or two B boxes, and one curly helix domain (Fig. 4A). To reveal the domain through which TRIM21 binds to NCAPH, we cotransfected plasmids containing TRIM21 domain mutants with flag-tagged NCAPH into 293T cells and observed their interaction with NCAPH. The results showed that when the ΔPRY/SPRY or CC domain was mutated, TRIM21 bound poorly to NCAPH, while other domains did not have an obvious influence on this interaction (Fig. 4B). In contrast, when we conducted co-IP experiments with antibodies labeled with HA, similar results were obtained (Fig. 4C). Thus, TRIM21 could bind to the NCAPH protein through its ΔPRY/SPRY and CC domains.

### TRIM21 promoted the degradation of the NCAPH protein through the ubiquitin proteasome pathway

We next treated cells with CHX and compared the effects of TRIM21 interference on the stability of the NCAPH protein. The results of the control group showed that the expression of NCAPH decreased with time. However, after interference with TRIM21, the level of the NCAPH protein significantly increased (Fig. 4D–G). Consistent with this finding, overexpression of TRIM21 resulted in a significant decrease in NCAPH protein levels (Fig. 4H). However, when cells were treated with pcDNA-3.1-TRIM21 and MG132 simultaneously, the level of NCAPH protein increased significantly. Thus, the inhibition of the proteasome pathway weakens the negative regulatory effect of TRIM21 on NCAPH protein levels (Fig. 4H). TRIM21 promoted the degradation of the NCAPH protein through the ubiquitination proteasome pathway.

### TRIM21 promoted the degradation of NCAPH by mediating K11-linked ubiquitination

To elucidate the mechanism by which TRIM21 regulates NCAPH, we transfected siTRIM21 and the HA-labeled ubiquitination plasmid HA-UB into HeLa and SiHa cells, respectively. Immunoprecipitation revealed that after interference with TRIM21, the ubiquitination level of the NCAPH protein decreased, while its protein level increased significantly (Fig. 4I). This finding suggested that TRIM21 mediates the ubiquitination of NCAPH, thus affecting its protein level. To further clarify the specific site at which NCAPH is modified by TRIM21, we added a specific site, the ubiquitin chain, to cells cocultured with cervical cancer cells. After interfering with TRIM21, the ubiquitination levels of NCAPH at K48 and K63 did not change significantly. In contrast, the level of ubiquitination at K11 decreased significantly. This result suggested that TRIM21 promoted NCAPH degradation by mediating K11-linked ubiquitination (Fig. 4J, K).

### TRIM21 mediated the ubiquitination of NCAPH through its RING domain

It is well known that the RING domain of the TRIM family is responsible for the E3 activity of TRIM proteins. To determine whether the RING domain of the TRIM21 protein mediates the degradation of NCAPH in cervical cancer, we transfected HeLa and SiHa cells with a full-length TRIM21 plasmid (HA-TRIM21) and a RING domain-mutated plasmid (TRIM21 ΔRING). Mutation of the RING domain resulted in the loss of regulation of NCAPH expression (Fig. 4L). This finding suggested that TRIM21 regulates NCAPH expression through its E3 ubiquitin ligase activity. Moreover, with the overexpression of TRIM21, the ubiquitination level of NCAPH significantly increased, while when the RING domain was mutated, its level significantly decreased and returned to the baseline level (Fig. 4M, N). Thus, the RING domain plays an important role in the TRIM21-mediated ubiquitination of NCAPH.

### TRIM21 promoted autophagy in cervical cancer cells by enhancing the formation of autophagosomes

**Fig. 5:**
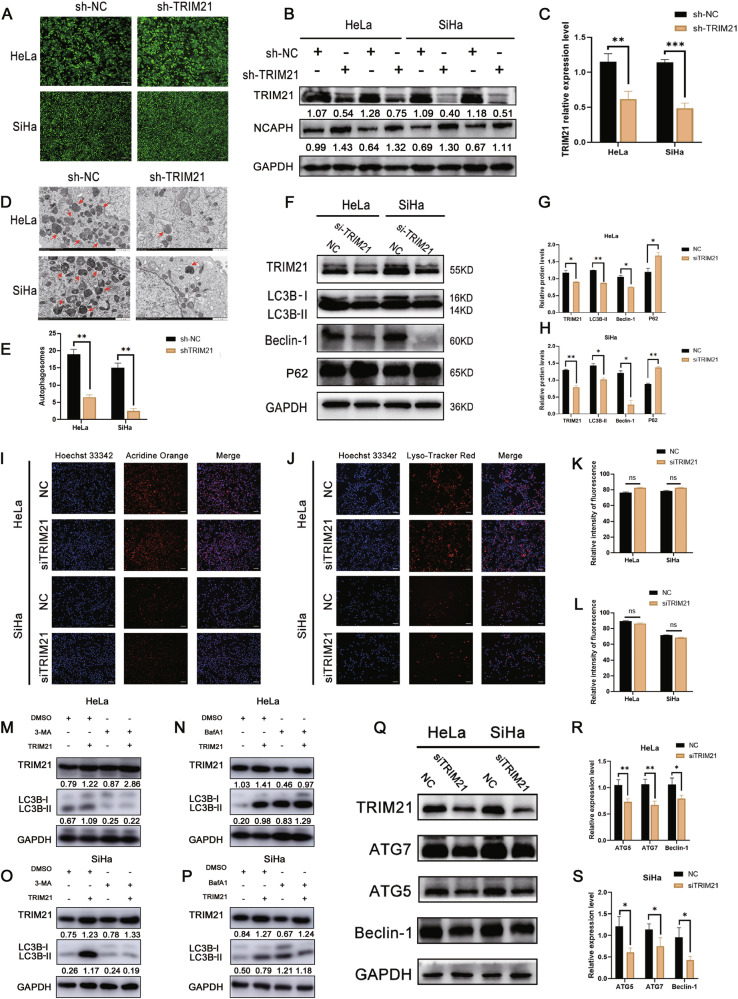
Interference with TRIM21 suppressed autophagy levels by inhibiting the formation of autophagosomes. **A**–**C** Construction of a cervical cancer cell line with stable knockout of TRIM21. HeLa and SiHa cells were transfected with lentivirus for 72 h and then observed under a fluorescence microscope. The cells were screened with puromycin, and the knockdown efficiency was further confirmed by western blotting. Scale = 100 µM. **D**, **E** Cervical cancer cells were treated with 5 µM rapamycin for 2–4 h and then with 20 µM chloroquine for 12 h and then observed by transmission electron microscopy. The number of autophagosomes was calculated by quantitative analysis. Results showed that silencing TRIM21 significantly decreased the number of autophagosomes in HeLa and SiHa cells. Data are representative images and means ± SD of 3 fields (100 μm^2^ per field). **F**–**H** Western blotting analysis showed that TRIM21 silencing decreased the expression of LC3B II and beclin-1 while increasing that of P62. **I**–**L** Cervical cancer cells were transfected with TRIM21 siRNA and then stained with AO and LysoTracker Red working solution. Hoechst 33342 (blue) was used to stain the nucleus. Statistical analysis revealed that knocking down TRIM21 had no effect on the fluorescence signals. **M**–**P** Western blotting analysis showed that 3-MA (2 mM) treatment almost completely reversed the changes in LC3B II caused by TRIM21 overexpression. In contrast, bafilomycin A1 (100 nM) treatment did not reverse the increase in LC3 II expression caused by TRIM21 overexpression. **Q**–**S** Western blotting analysis showed that knocking down TRIM21 expression significantly decreased the expression levels of autophagosome formation-related proteins (Beclin-1, ATG5, and ATG7). shTRIM21 and siTRIM21, TRIM21 interference group; shNC and siNC, negative control group. TRIM21, pcDNA3.1-TRIM21 plasmid; pcDNA3.1, mock vector. Scale = 25 μM. *P < 0.05, **p < 0.01.

To examine whether TRIM21 could regulate autophagy, we transfected HeLa and SiHa cells with lentivirus containing TRIM21 shRNA. After transfection, the intensity of the green fluorescence signal in the cells increased significantly (Fig. 5A). Western blotting analysis revealed that when the expression of TRIM21 decreased, that of NCAPH increased significantly. This confirmed the successful construction of the cell lines (Fig. 5B, C). TEM assay revealed that compared with the NC group (19.000 ± 0.577 per field for HeLa; 15.667 ± 0.882 per field for SiHa), stable elimination of TRIM21 significantly reduced the number of autophagosomes in HeLa (6.667 ± 0.333 per field) and SiHa cells (2.667 ± 0.333 per field) (*p* < 0.05) (Fig. 5D, E). Furthermore, with decreasing TRIM21 expression, the expression of LC3 and beclin-1 decreased, while that of P62 increased significantly (Fig. 5F–H).

To clarify the mechanism by which TRIM21 regulates autophagy, we performed acridine orange (AO) and lysosomal tracker red staining. The fluorescence of cells treated with TRIM21 siRNA was not significantly different from that of cells treated with NC siRNA (Fig. 5I–L). This suggested that TRIM21 had no significant effect on the autophagy-mediated lysosomal fusion process. Western blotting analysis revealed that TRIM21 overexpression significantly increased the level of LC3 II in HeLa and SiHa cells, and 3-MA treatment reversed the changes in the ratio of LC3 II caused by TRIM21 overexpression. Conversely, BafA1 treatment did not reverse the changes in the LC3 II ratio caused by the overexpression of TRIM21 (Fig. 5M–P). Furthermore, after interference with TRIM21, the expression of ATG5, ATG7, and beclin-1 decreased significantly (Fig. 5Q–S). These results suggested that TRIM21 is an essential protein involved in the promotion of autophagy and can promote the process of autophagy in cervical cancer cells.

### TRIM21 promoted autophagy in cervical cancer cells through NCAPH

**Fig. 6:**
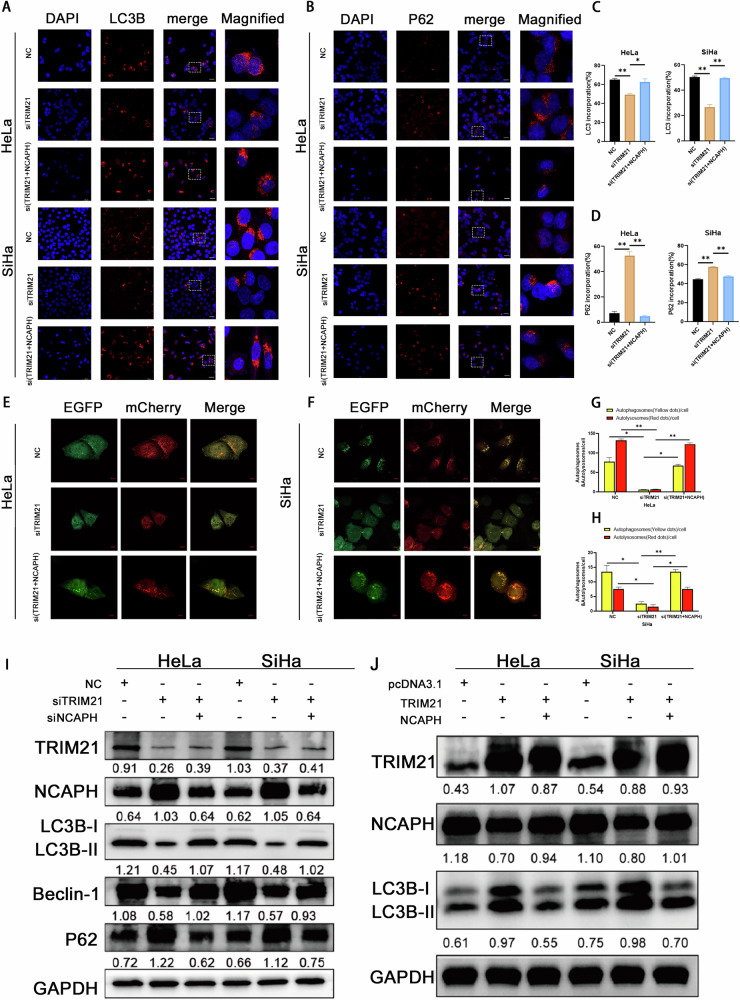
TRIM21 promotes autophagy in cervical cancer cells through NCAPH. **A**–**D** Immunofluorescence staining was used to detect changes in the expression of LC3B and P62 in HeLa and SiHa cells. Interference with TRIM21 decreased the LC3B signal but increased the P62 signal. Knocking down NCAPH significantly reversed the changes caused by siTRIM21. Scale bar = 50 µm. **E**–**H** The mRFP-GFP-LC3 indicator system showed that interference with TRIM21 significantly decreased the spot ratio of mRFP to GFP, while silencing NCAPH dramatically reversed the changes in the spot ratio caused by siTRIM21 treatment. The autophagic flow process was observed and analyzed via confocal microscopy. ImageJ software was used to calculate the number of yellow and red puncta. Scale bar, 10 µm. **I**, **J** Western blotting analysis showed that interference with NCAPH eliminated the changes caused by knocking down TRIM21 (**I**). In contrast, overexpression of NCAPH eliminated the increase in LC3B II levels caused by overexpression of TRIM21 (**J**). The figure shows the representative results of three experiments. *P < 0.05, **p < 0.01, ***p < 0.001.

To confirm whether TRIM21 regulates autophagy through NCAPH, we performed immunofluorescence staining. The results showed that in HeLa and SiHa cells, interference with TRIM21 resulted in a significant decrease in the LC3B signal, while the P62 signal was significantly enhanced. In the rescue experiment, knocking down NCAPH significantly reversed the changes in LC3B and P62 signals caused by interference with TRIM21 (Fig. 6A–D). Similarly, the mRFP-GFP-LC3 indicator system showed that interference with TRIM21 significantly decreased the spot ratio of mRFP and GFP, while silencing NCAPH dramatically reversed the changes in the spot ratio caused by siTRIM21 treatment (Fig. 6E–H). Consistently, siTRIM21 treatment increased the expression of NCAPH and P62 but significantly decreased that of LC3B and beclin-1 (Fig. 6I). Interference with NCAPH eliminated the changes caused by interference with TRIM21 (Fig. 6I). In contrast, overexpression of NCAPH eliminated the increase in LC3 levels caused by overexpression of TRIM21 (Fig. 6J). These results confirmed that TRIM21 promoted autophagy in cervical cancer cells by downregulating the expression of NCAPH.

### NCAPH promoted the proliferation of cervical cancer cells by inhibiting autophagy

**Fig. 7:**
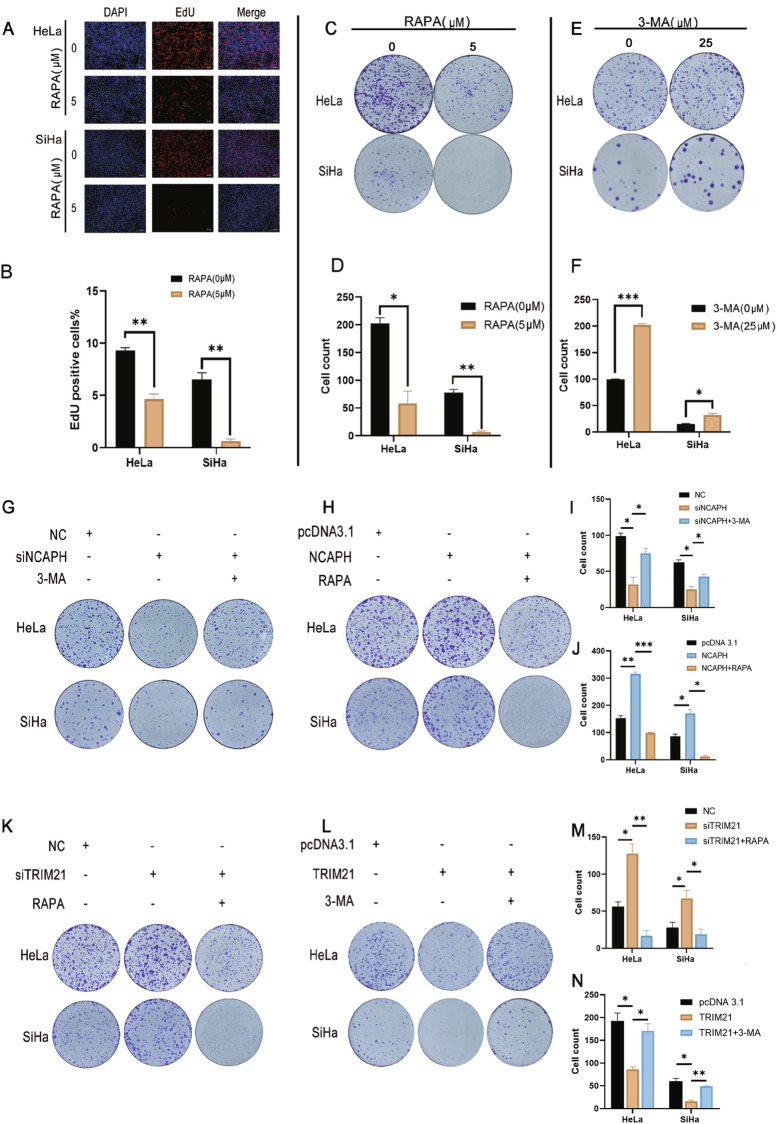
NCAPH and TRIM21 could regulate the proliferation of cervical cancer cells through autophagy. **A**, **B** HeLa and SiHa cells were treated with RAPA (5 μM) for 24 h, and the EdU assay was used to detect cell proliferation capacity. **C**–**F** HeLa and SiHa cells were treated with RAPA (5 μM) or 3-MA (25 μM) for 24 h, and colony formation experiments were used to evaluate colony formation ability. **G**, **I** HeLa and SiHa cells were transfected with siNCAPH alone or in combination with 3-MA (25 μM). A colony formation assay was used to determine whether 3-MA could rescue the siNCAPH-induced reduction in proliferation. **H**, **J** HeLa and SiHa cells were transfected with the NCAPH overexpression plasmid alone or in combination with RAPA (5 μM). A colony formation assay was used to assess whether RAPA could rescue the increase in proliferation induced by ectopic NCAPH expression. **K**, **M** HeLa and SiHa cells were transfected with siTRIM21 alone or in combination with RAPA (5 μM) before the colony formation assay was performed. **L**, **N** HeLa and SiHa cells were transfected with the TRIM21 overexpression plasmid alone or treated in combination with 3-MA (25 μM) before performing the colony formation assay. The figure shows the representative results of three experiments. siTRIM21, TRIM21 interference group; NC, negative control group. TRIM21, pcDNA3.1-TRIM21 plasmid; pcDNA3.1, mock vector. *P < 0.05, **p < 0.01, ***p < 0.001.

To investigate whether autophagy affects the proliferation of cervical cancer cells, we treated cells with RAPA or 3-MA. The results showed that RAPA treatment significantly reduced proliferation (Fig. 7A–D), while treatment with 3-MA significantly improved proliferation (Fig. 7E, F). This suggested that autophagy could inhibit the proliferation of cervical cancer cells. To verify whether NCAPH promoted cell proliferation by inhibiting autophagy, we knocked down or overexpressed NCAPH in HeLa and SiHa cells and treated the cells with 3-MA or RAPA, respectively. The results showed that 3-MA treatment significantly reversed the decrease in proliferation caused by silencing NCAPH (Fig. 7G, I). Consistently, overexpression of NCAPH improved the colony formation ability of cervical cancer cells, while the addition of RAPA significantly reduced the effects induced by overexpression of NCAPH (Fig. 7H, J). These results indicated that NCAPH promoted the proliferation of cervical cancer cells by inhibiting autophagy.

### TRIM21 promoted autophagy and inhibited the proliferation of cervical cancer cells through its regulation of NCAPH

We inhibited the expression of TRIM21 in cervical cancer cells and performed a colony formation assay with or without RAPA treatment. With the reduction in TRIM21 expression, the colony formation capacity of cervical cancer cells increased significantly, and with the addition of RAPA, it decreased dramatically (Fig. 7K, M). Consistent with this finding, overexpression of TRIM21 had the opposite effect (Fig. 7L, N). These results indicated that TRIM21 could inhibit cervical cancer cell proliferation by promoting autophagy.

**Fig. 8:**
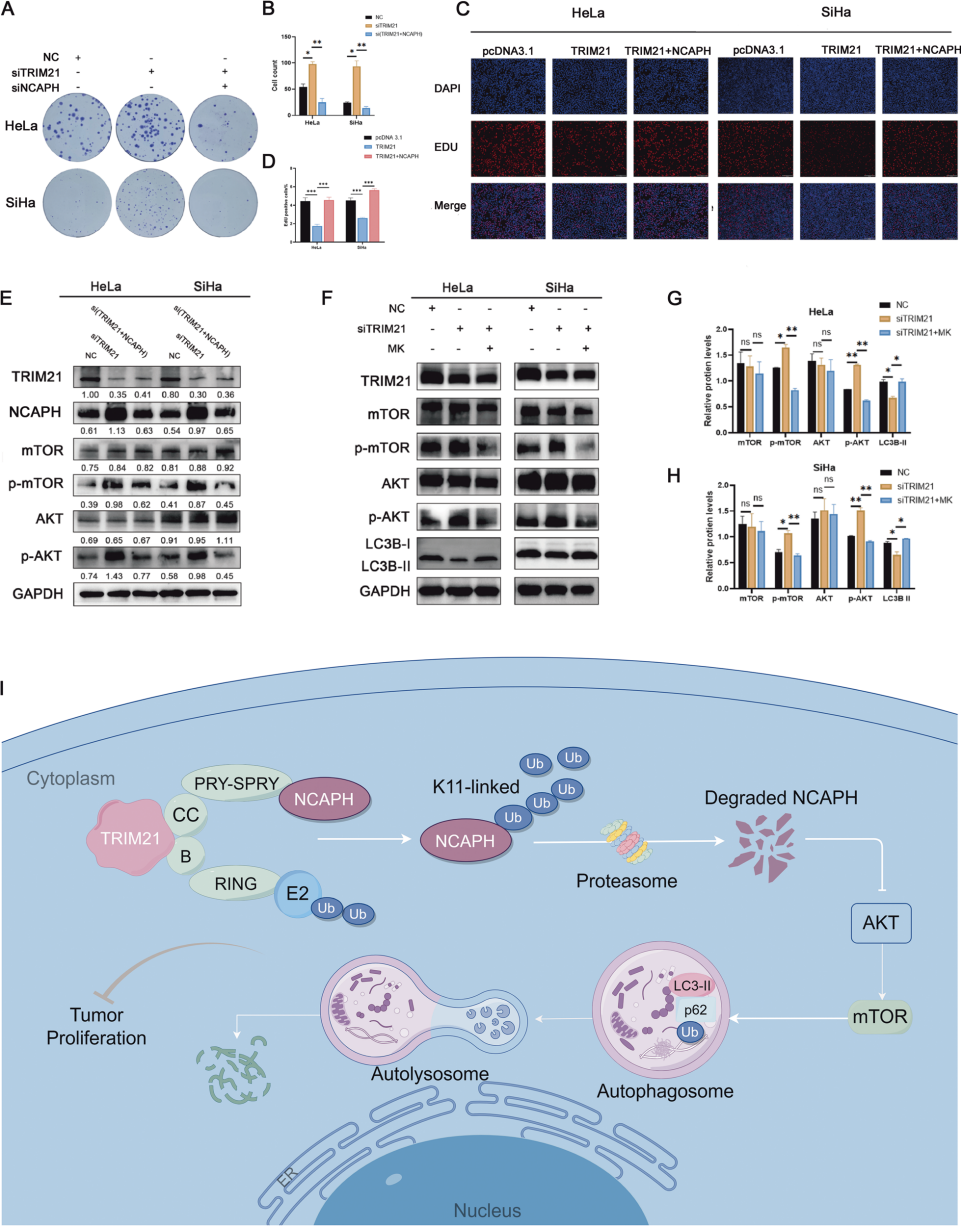
TRIM21 promotes autophagy and inhibits cell proliferation through NCAPH via an AKT/mTOR-dependent mechanism. **A**, **B** Silencing TRIM21 significantly increased the number of colonies of cervical cancer cells, while interference with NCAPH dramatically reversed the changes in colony numbers induced by siTRIM21. **C**, **D** The EdU assay was used to detect changes in cell proliferation capacity. Ectopic expression of NCAPH rescues the reduction in cell proliferation induced by the increase in TRIM21 expression. **E** Cells were treated with siTRIM21 alone or in combination with siNCAPH. Interference with TRIM21 expression increased p-AKT and p-mTOR protein levels but had no significant effect on total AKT or mTOR levels. NCAPH suppression reverses the effects of siTRIM21 on the protein levels of p-AKT and p-mTOR. **F**–**H** Western blotting analysis showing that treatment with an AKT/mTOR pathway inhibitor (MK2206) reversed the effects of TRIM21 silencing on the expression of p-AKT, p-mTOR and LC3B. **I** The proposed model in the study. NCAPH, which is ubiquitinated by TRIM21, promotes cell proliferation by inhibiting autophagy in cervical cancer through AKT/mTOR-dependent signaling. The figure shows the representative results of three experiments. *P < 0.05, **p < 0.01, ***p < 0.001.

Moreover, the colony formation assay showed that cell proliferation was significantly enhanced after transfection with siTRIM21, while cotransfection with siNCAPH significantly reversed the increase in proliferation caused by siTRIM21 (Fig. 8A, B). Consistently, an EdU assay revealed that overexpression of NCAPH significantly reversed the decrease in proliferation caused by overexpression of TRIM21 (Fig. 8C, D). The data indicated that TRIM21 promoted autophagy and inhibited cell proliferation by inhibiting NCAPH.

### TRIM21 activated autophagy by inhibiting NCAPH and its downstream AKT/mTOR pathway

The AKT/mTOR pathway is a key molecular signaling pathway that plays an essential role in the regulation of autophagy. Previously, we demonstrated that NCAPH can promote the activation of the AKT/mTOR pathway [23]. However, it is unknown whether TRIM21 can regulate the AKT/mTOR pathway in cervical cancer. Next, we investigated whether TRIM21 could regulate the AKT/mTOR pathway via NCAPH. The results showed that interference with TRIM21 significantly increased the levels of phosphorylated AKT and mTOR but had no significant effect on the total AKT or mTOR protein levels (Fig. 8E). This finding suggested that TRIM21 could inhibit the activation of the AKT/mTOR pathway. Moreover, silencing NCAPH dramatically eliminated the activation of the AKT/mTOR pathway induced by silencing TRIM21 (Fig. 8E). Therefore, TRIM21 could inhibit the AKT/mTOR pathway by inhibiting NCAPH. Furthermore, treatment with MK-2206 (an AKT/mTOR pathway inhibitor) reduced the upregulation of p-AKT and p-mTOR and eliminated the decrease in autophagy protein levels induced by interference with TRIM21 (Fig. 8F–H). Therefore, TRIM21 promoted autophagy in cervical cancer by inhibiting NCAPH and the downstream AKT/mTOR pathway.

### Coexpression of TRIM21, NCAPH, and autophagy-related proteins in vivo

To further verify the correlation between TRIM21, NCAPH and autophagy, we performed a bioinformatics analysis in cervical cancer. cBioportal and GEPIA analyses revealed that NCAPH mRNA expression was negatively correlated with that of LC3 and TRIM21 but positively correlated with that of SQSTM1 (Supplementary Fig. S2A–C). Similar results were observed in other solid tumors, such as esophageal squamous cell carcinoma and gastric cancer (data not shown). This finding suggested that NCAPH is involved in the regulation of autophagy in human malignancies. To further confirm these results, we performed immunohistochemical staining and found that TRIM21 was overexpressed in 129 patients with cervical cancer (129/220, 58.64%), NCAPH was overexpressed in 84 patients (84/220, 38.18%), and beclin-1 was overexpressed in 156 patients (156/220, 70.91%) (Supplementary Fig. S2D). The correlations between TRIM21 expression and NCAPH (r = −0.5748), NCAPH and beclin-1 (r = −0.5678), and TRIM21 and beclin-1 (r = 0.5188) were statistically significant (all *p* values < 0.001).

## Discussion

Autophagy abnormalities are known to exist in approximately half of human malignancies, and overactivated autophagy can lead to cell death [24]. During the progression of normal cervix to invasive cancer, the expression of key autophagy markers (LC3 and beclin-1) gradually decreases and is negatively correlated with tumor differentiation, lymph node metastasis, and patient prognosis [25, 26]. Autophagy can also inhibit malignant growth, the stemness of tumor stem cells and tumorigenicity in nude mice with cervical cancer [27–30]. Park et al. revealed that autophagy could limit the progression of cervical cancer by mediating the degradation of TNF receptor-related factor 6 (TRAF6) [31]. Yang et al. reported that beclin-1 overexpression could improve anticancer drug sensitivity [32], while the combination of cisplatin and graphene oxide could increase tumor cell death by inducing autophagy, thus achieving chemosensitivity [33]. Therefore, abnormal autophagy is an important factor in promoting the progression of cervical lesions, and autophagy-targeted therapies are expected to improve the effects and prognosis of patients. However, the study of cervical cancer autophagy is relatively limited.

In the present study, we demonstrated that blocking NCAPH expression had no effect on the cell cycle, apoptosis, or aging of cervical cancer cells. However, interference with NCAPH expression significantly increased autophagic body formation and promoted the autophagic flow process in cervical cancer. Therefore, NCAPH is a novel autophagy-associated protein that plays an important role in the regulation of autophagy in cervical cancer. The autophagy process includes a series of complex processes, such as autophagosome formation, autophagosome-lysosome fusion, and autophagic lysosome degradation [34]. We found that NCAPH had no effect on the function of lysosomes. It affects the initial stage of autophagy, that is, the formation of autophagic bodies and vesicles. The autophagy process is regulated by different genes related to autophagy (ATG), which participate in different stages of autophagy [34]. The number of LC3-II cleavage products is related to the degree of autophagic body formation. Beclin-1 promotes the localization of autophagic proteins in autophagic vesicles. ATG7 can regulate the binding of ATG12 and ATG5 to form a protein complex that can be transported to the membrane to form autophagic vesicles [34]. Similarly, NCAPH can regulate proteins involved in the initial stage of autophagy, such as LC3 II, beclin-1, ATG5, and ATG7. Therefore, the regulatory role of NCAPH in autophagy is achieved mainly through its regulation of the stage of autophagic body formation in cervical cancer cells.

NCAPH is a newly identified oncoprotein, and the spectrum of host proteins that interact with it is not yet clear. Here, we found that NCAPH was a novel target protein of the E3 ligase TRIM21 in cervical cancer. TRIM21, a member of the TRIM family, regulates cell apoptosis, the cell cycle, and differentiation through its ubiquitination of various substrates [35]. It combines with the substrate through the B box and the CC region and recruits the ubiquitin ligase E2 from the RING domain to approach the substrate and ubiquitinate it [35]. Consistent with these findings, we demonstrated that TRIM21 combines with NCAPH through its ΔPRY/SPRY and CC domains and mediates the degradation of NCAPH through its RING domain. The ubiquitin monomers have 7 lysine residues and 1 methionine residue (M1). Different ubiquitination modifications regulate different cellular functions [36–39]. For example, TRIM21 can promote K27-linked polyubiquitination of the mitochondrial antiviral signaling protein (MAVS) to positively regulate the innate immune response [40]. TRIM21 can also catalyze the formation of K48-linked ubiquitin moieties to degrade and inhibit viruses [41]. However, the substrate and function of K11-linked ubiquitin mediated by TRIM21 have rarely been reported. This study revealed that TRIM21 could degrade the NCAPH protein through K11-linked ubiquitination. Previous studies have reported that TRIM21 can reduce the stability of autophagy-associated proteins that influence the autophagy process [42] or that it can act directly as an autophagic receptor to recognize and recruit target proteins for highly selective autophagy [43, 44]. However, its relationship with autophagy in cervical cancer is unknown. In our study, we elucidated that TRIM21 could inhibit autophagy by regulating the expression of NCAPH in cervical cancer cells. Similar with NCAPH, its regulation promoted the formation of autophagosomes. Consistent with in vitro findings, TRIM21 expression was negatively correlated with that of NCAPH, but positively with Beclin-1 in cervical cancer patients. Unexpectedly, TRIM21 was not significantly associated with the prognosis of patients (data not shown). We speculate that this is due to the limited number of cases enrolled, and weakened prognostic value of TRIM21 caused by the complex regulatory effect of TRIM21 on host intracellular substrates. These results confirmed the mechanism underlying the interaction between TRIM21 and NCAPH and revealed for the first time the regulatory effect of TRIM21-mediated K11 ubiquitination on autophagy.

Autophagy can inhibit the generation of various tumors by increasing chromosome stability and reducing cell survival, metastasis, and metabolic and oxidative stress. Recent studies have shown that autophagy can also regulate the proliferation ability of different carcinomas, indicating a close association between autophagy and cell proliferation [45–47]. Consistently, we found that TRIM21 promoted autophagy and reduced cell proliferation by controlling NCAPH expression. The AKT/mTOR pathway plays an important role in the regulation of cervical cancer cell growth [48]. TRIM21 has tight crosstalk with the AKT/mTOR pathway. Lin et al. reported that TRIM21 mediated RNF1 degradation via ubiquitination, thus alleviating the negative regulation of RNF1 in the AKT/mTOR pathway in breast cancer [49]. However, Chen’s study showed that activation of the AKT/mTOR pathway inhibited TRIM21 expression and relieved its degradation of G6PD [50]. Therefore, the regulatory role of TRIM21 in the AKT/mTOR pathway remains controversial and needs further research. Here, we demonstrated that TRIM21 promoted autophagy and reduced proliferation by degrading the NCAPH protein and inhibiting the downstream AKT/mTOR pathway. Our data demonstrated a novel molecular pathway in cervical cancer, that is, TRIM21 degrades NCAPH through K-11 ubiquitination, thus inhibiting the activation of the downstream AKT/mTOR pathway, promoting autophagosome formation and inhibiting cell proliferation.

## Conclusions

Previously, we identified NCAPH as a novel oncoprotein involved in the occurrence and development of cervical cancer. Here, we showed that TRIM21 was the upstream regulatory protein of NCAPH and demonstrated the detailed mechanism of this interaction. Furthermore, we elucidated that the TRIM21-NCAPH axis increased the level of autophagy and reduced the cell proliferation rate by inhibiting the AKT/mTOR signaling pathway (Fig. 8I). Overall, these results deepen our understanding of the role of NCAPH and TRIM21 in autophagy, enrich the study of the carcinogenic mechanism of NCAPH, and provide a potential rationale for developing a treatment strategy targeting autophagy in the future. In recent years, anticancer drugs targeting E3 ubiquitin ligases have attracted increased amounts of attention. Compared to proteasome inhibitors such as bortezomib, these drugs have the advantages of high selectivity and mild side effects. Therefore, the future development of TRIM21-targeting agonists is expected to significantly inhibit cervical cancer growth.

### Supplementary information

Supple Fig1

Supple Fig2

Supplementary legends

Raw data(Full and uncropped western blots)

## Data Availability

All data supporting this study are presented in this published article and in its Supplementary information files.
