# Supplementary figures and images for: NCAPH, ubiquitinated by TRIM21, promotes cell proliferation by inhibiting autophagy of cervical cancer through AKT/mTOR dependent signaling
Source: Cell Death Dis. 2024 Aug 6;15(8):565. doi: 10.1038/s41419-024-06932-y (PMC11300717; doi:10.1038/s41419-024-06932-y)

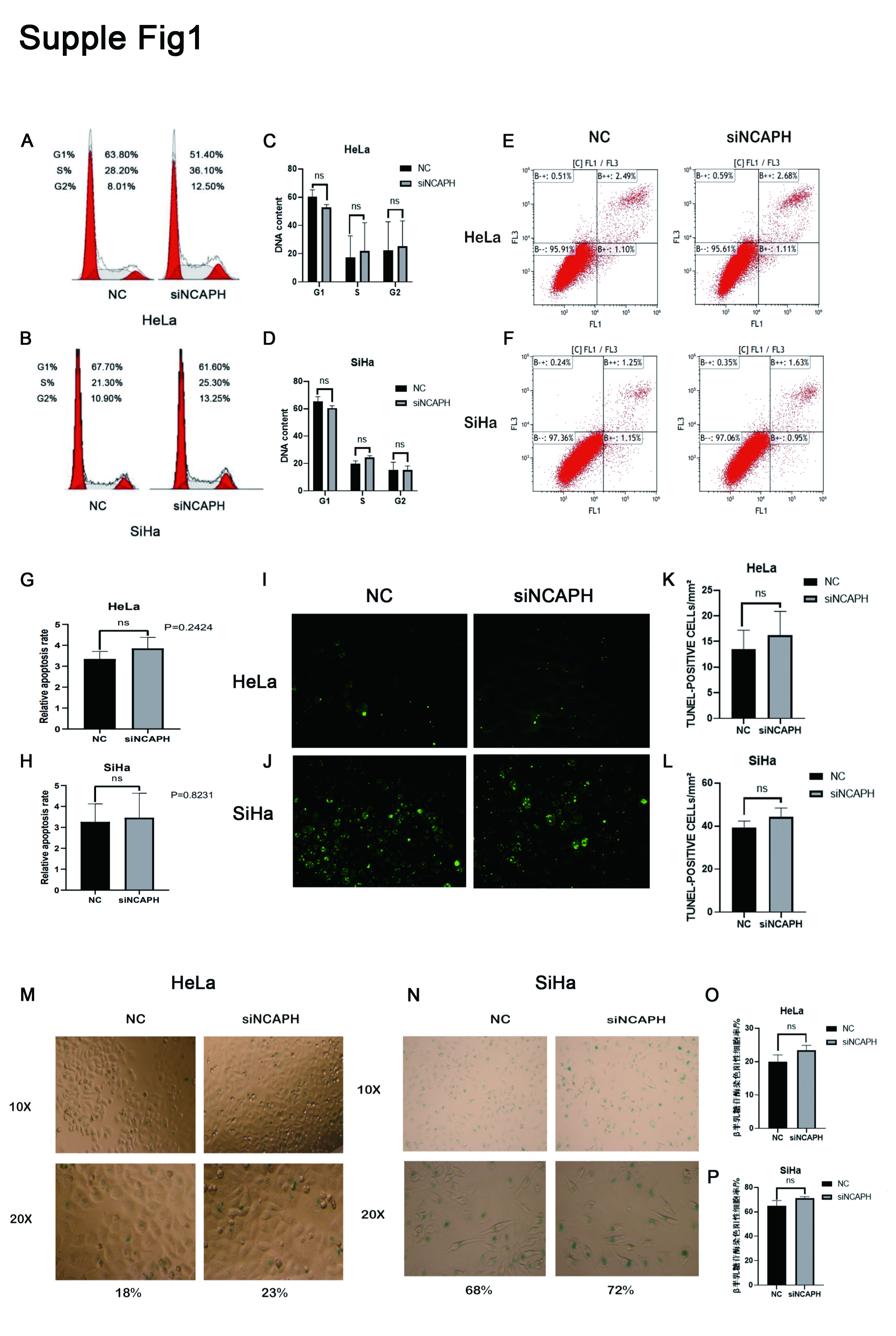

Supplement: Supplementary file 1 — Supple Fig1 [file 41419_2024_6932_MOESM1_ESM.tif]

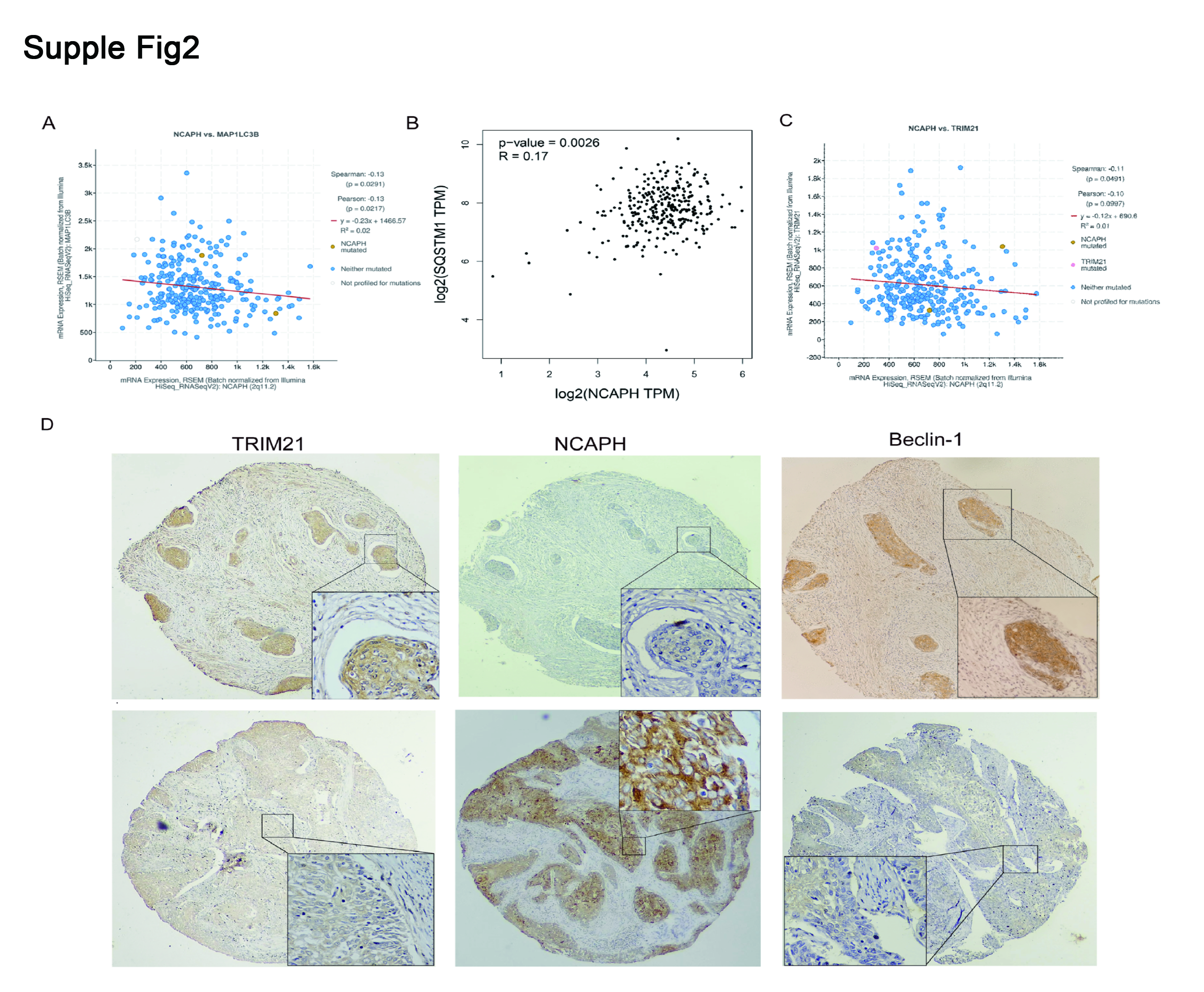

Supplement: Supplementary file 2 — Supple Fig2 [file 41419_2024_6932_MOESM2_ESM.tif]
