# Supplementary material for: NCAPH, ubiquitinated by TRIM21, promotes cell proliferation by inhibiting autophagy of cervical cancer through AKT/mTOR dependent signaling
Source: Cell Death Dis. 2024 Aug 6;15(8):565. doi: 10.1038/s41419-024-06932-y (PMC11300717; doi:10.1038/s41419-024-06932-y)
Supplement: Supplementary file 3 — Supplementary legends [file 41419_2024_6932_MOESM3_ESM.docx]

**Suppl. Figure S1. Effects of NCAPH on the cell cycle, apoptosis, and aging of cervical cancer cells**

(A-D) Flow cytometry was used to detect the effects of NCAPH interference on the cell cycle of cervical cancer cells. Compared with the control group (NC), the NCAPH-silenced group (siNCAPH) had a shorter G1 phase and longer S and G2 phases in HeLa and SiHa cells. However, the difference was not statistically significant. All *P* values >0.05. (E-L) Flow cytometry (E-H) and TUNEL apoptosis (I-L) were used to determine the effect of NCAPH interference on the apoptosis of HeLa and SiHa cells. Compared with the control group, the interference group exhibited a slight increase in the proportion of apoptotic cells. However, the difference was not statistically significant. All *p* values > 0.05. (M-P) The effects of NCAPH on HeLa and SiHa cell senescence were detected by β-galactosidase staining. Compared with those in the control group, no significant changes were observed between the control and siNCAPH groups in the aging population of HeLa and SiHa cells after interference with NCAPH.

**Suppl. Figure S2.** **The correlation of TRIM21, NCAPH, and autophagy in cervical cancer *in vivo*.**

(A-C) The cBioPortal (A, C) and GEPIA (B) datasets revealed associations between NCAPH, LC3, SQSTM1, and TRIM21 mRNA expression levels. (D) Immunohistochemical staining showing the associations of TRIM21, NCAPH, and beclin-1 with protein levels. The correlation between them was statistically significant (all *p* values <0.001). (E) Proposed model for the regulatory role of TRIM21 and NCAPH in the autophagy and proliferation of cervical carcinoma cells.
