# Supplementary material for: NCAPH, ubiquitinated by TRIM21, promotes cell proliferation by inhibiting autophagy of cervical cancer through AKT/mTOR dependent signaling
Source: Cell Death Dis. 2024 Aug 6;15(8):565. doi: 10.1038/s41419-024-06932-y (PMC11300717; doi:10.1038/s41419-024-06932-y)
Supplement: Supplementary file 4 — Raw data(Full and uncropped western blots) [file 41419_2024_6932_MOESM4_ESM.pdf]

Figure 1

Figure 1C

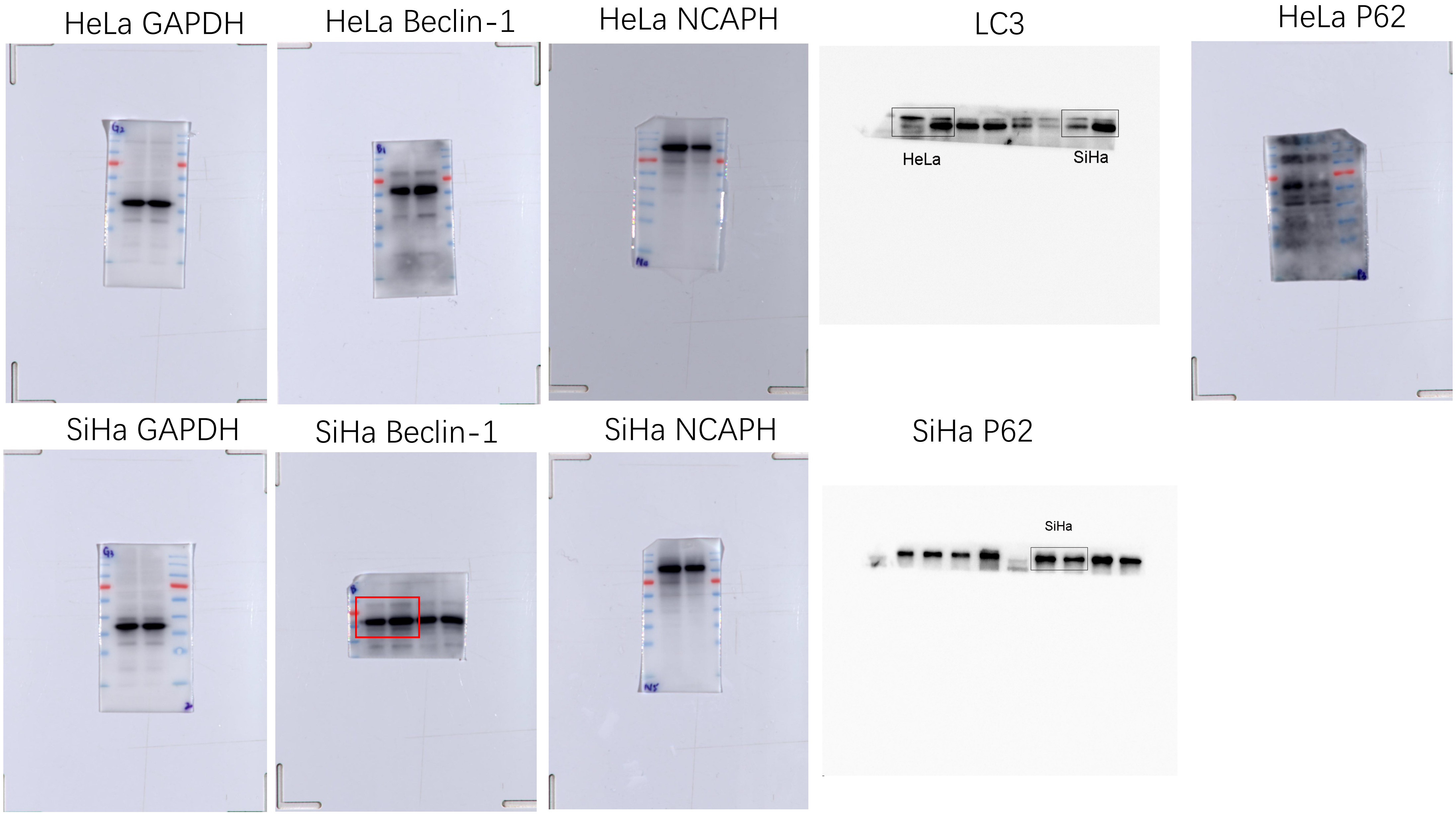

Figure 2

Figure 2E

HeLa GAPDH HeLa NCAPH HeLa LC3

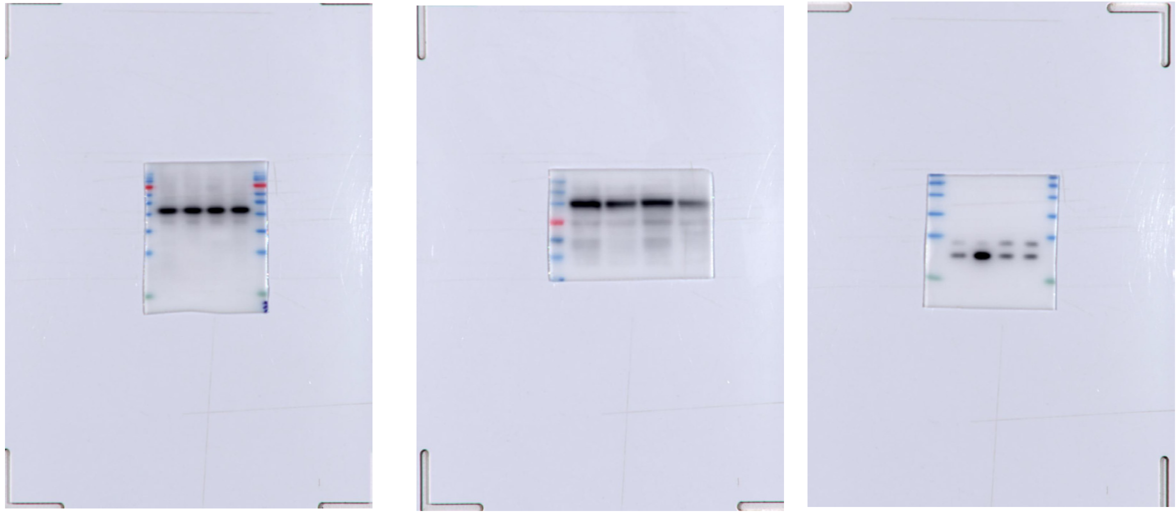

Figure 2F

HeLa GAPDH HeLa NCAPH HeLa LC3

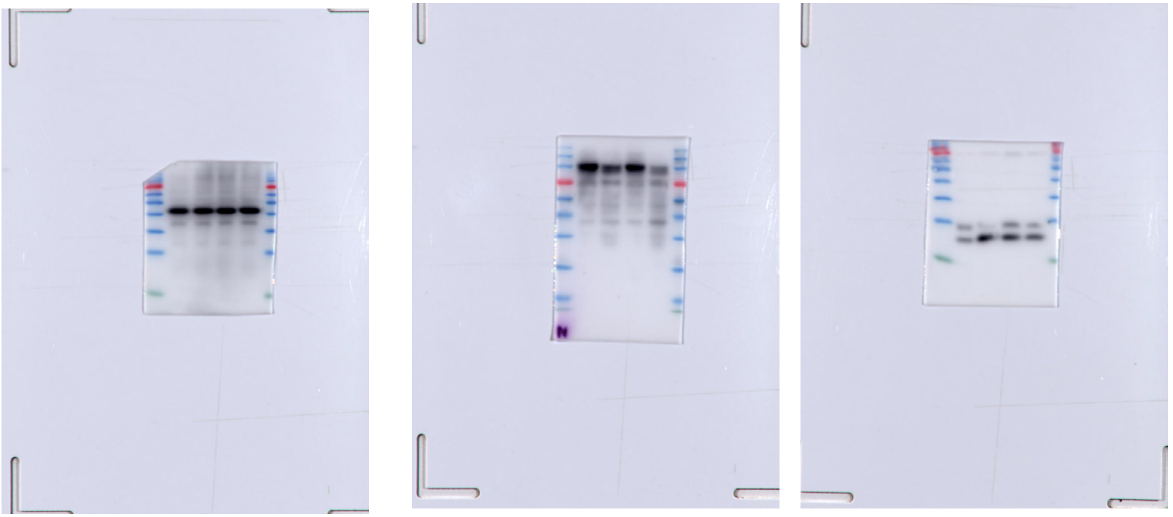

Figure 2G

SiHa GAPDH SiHa NCAPH SiHa LC3

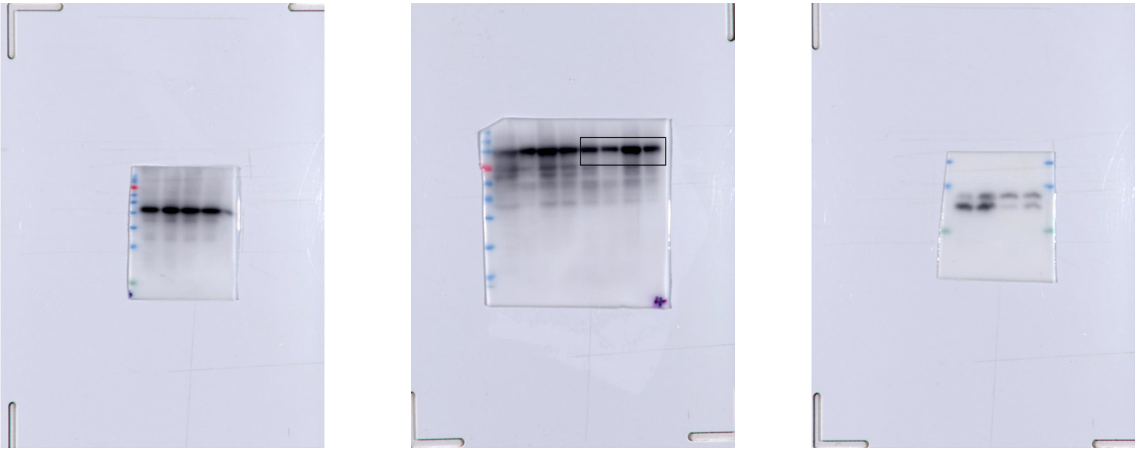

Figure 2H

SiHa GAPDH SiHa NCAPH SiHa LC3

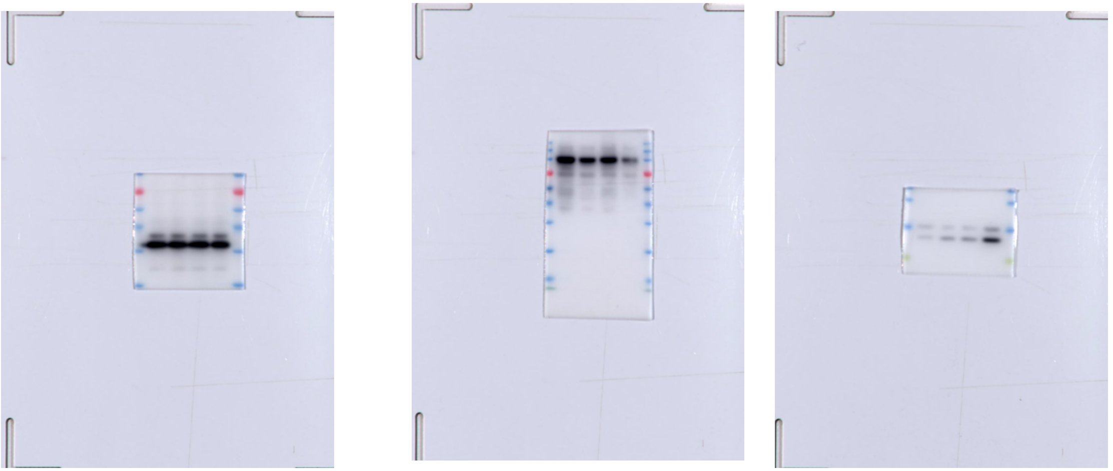

Figure 2
Figure 2I

HeLa+SiHa NCAPH

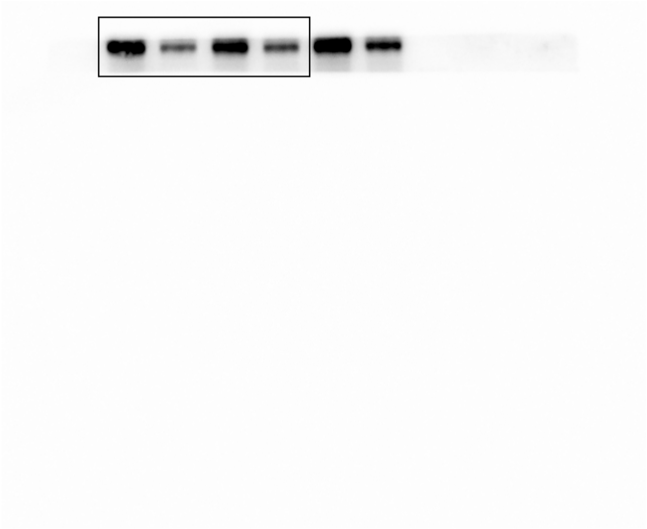

HeLa ATG7

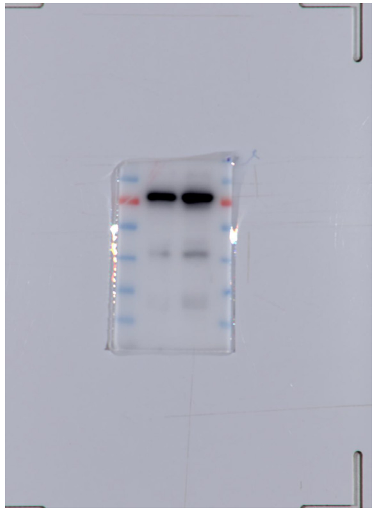

HeLa ATG5

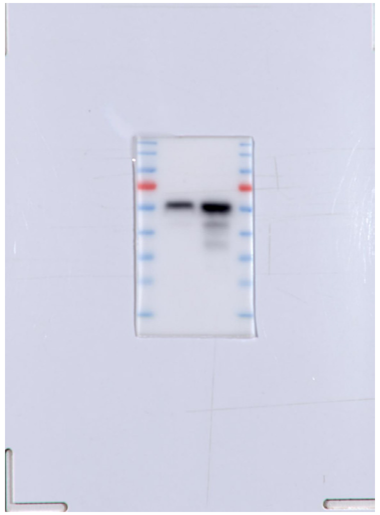

HeLa GAPDH

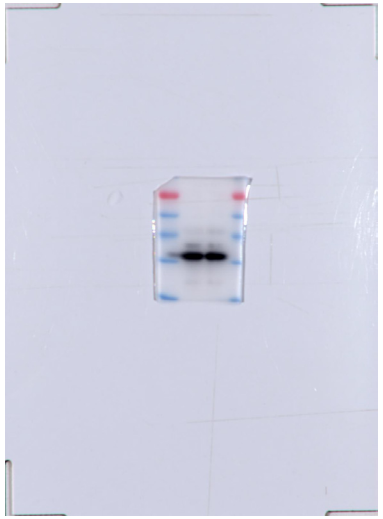

HeLa+SiHa Beclin-1

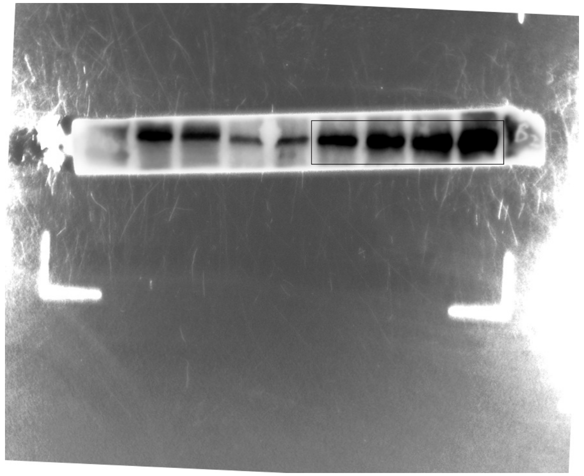

SiHa ATG7

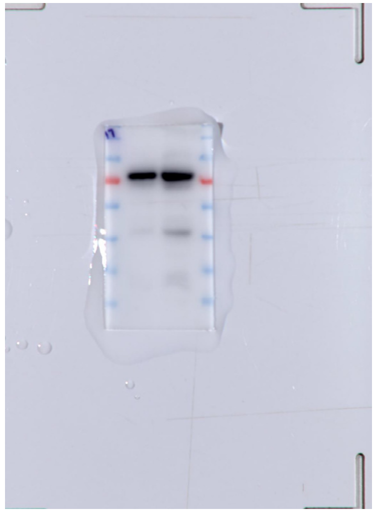

SiHa ATG5

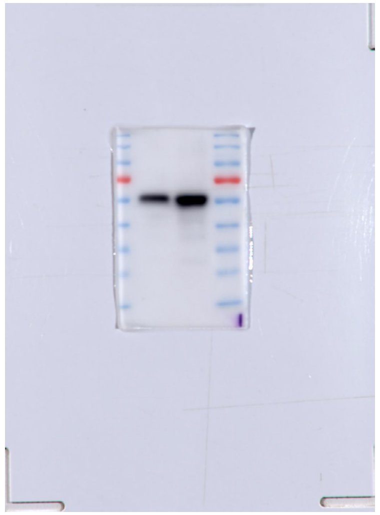

SiHa GAPDH

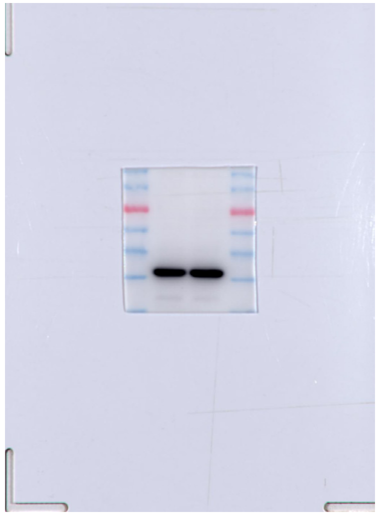

Figure 3

Figure 3A

HeLa GAPDH

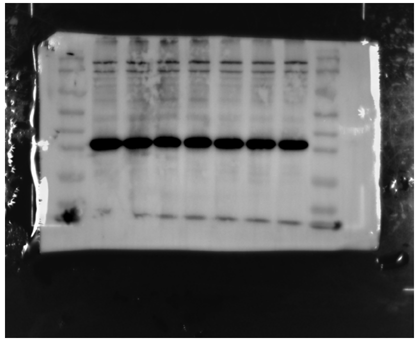

HeLa NCAPH

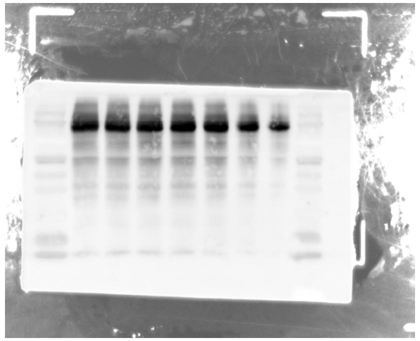

Figure 3B

SiHa GAPDH

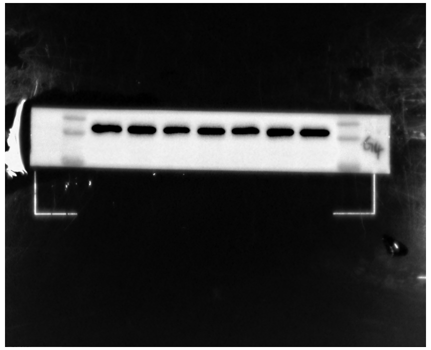

SiHa NCAPH

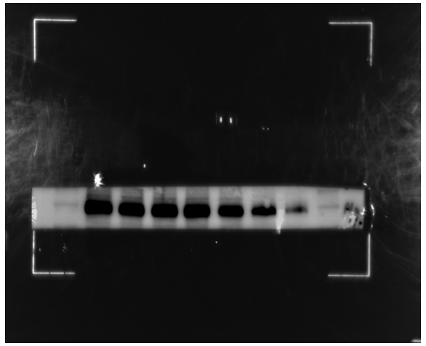

Figure 3D

HeLa+SiHa GAPDH

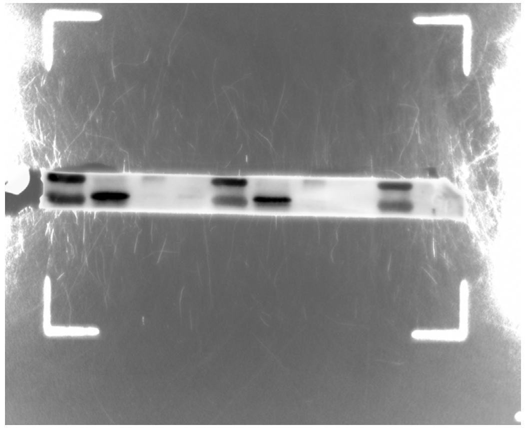

HeLa NCAPH

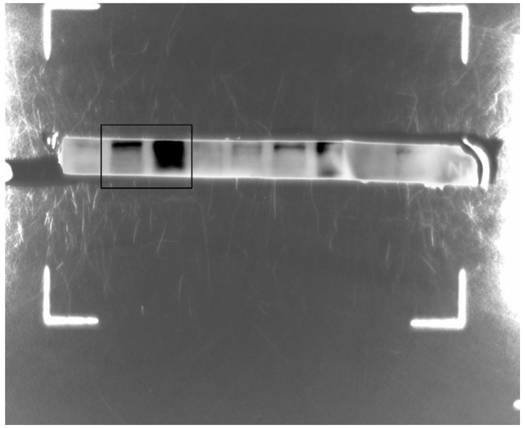

SiHa NCAPH

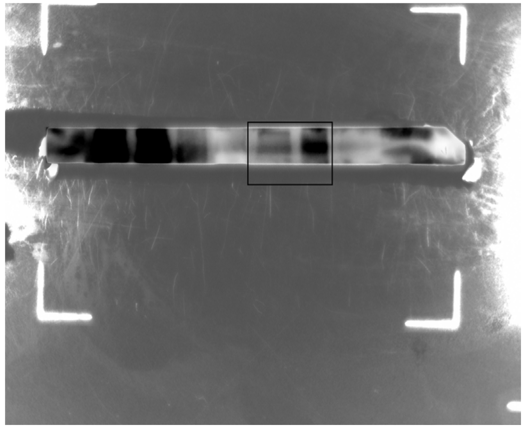

HeLa+SiHa TRIM21

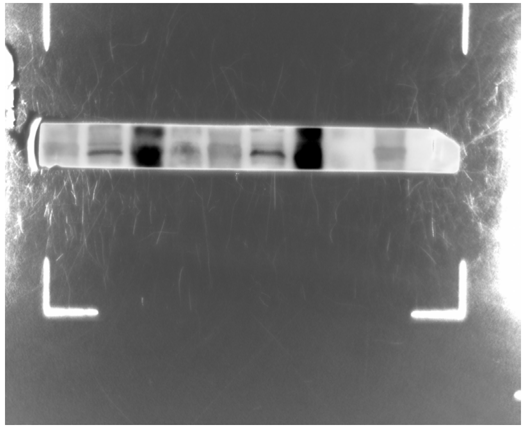

Figure 3

Figure 3E

HeLa+SiHa GAPDH

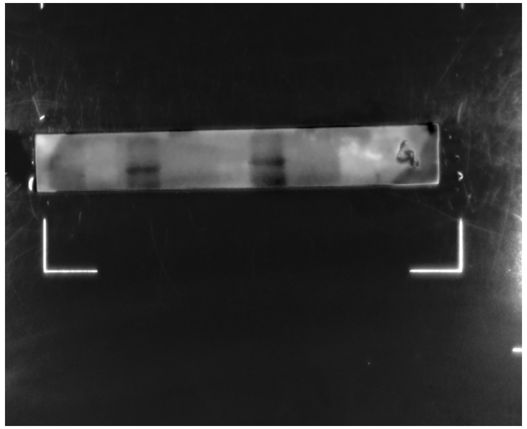

HeLa+SiHa NCAPH

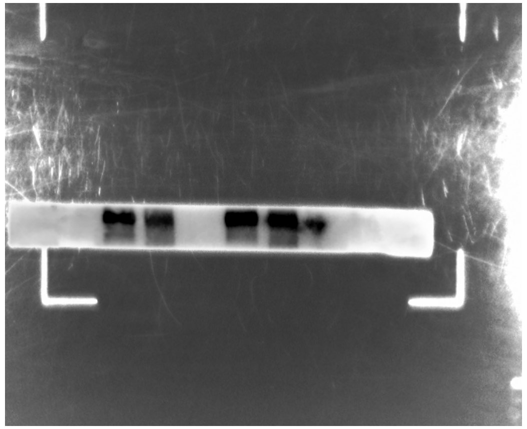

HeLa+SiHa TRIM21

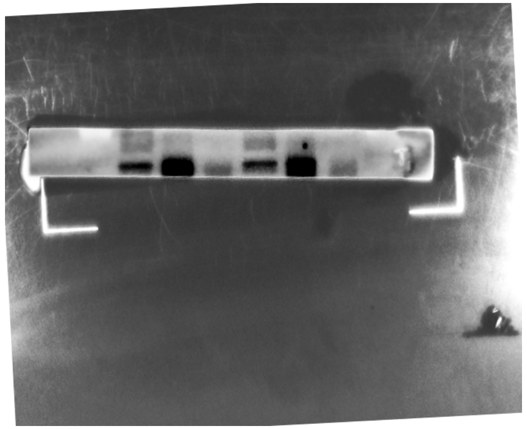

Figure 3G

GAPDH

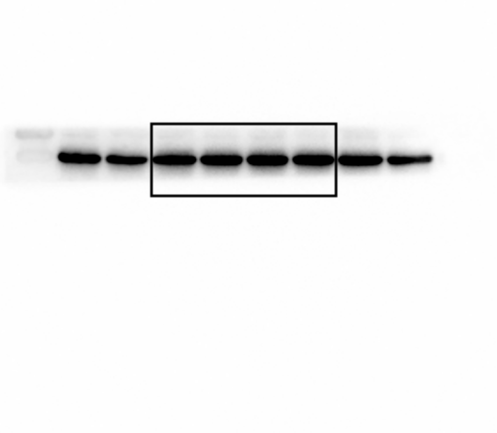

NCAPH

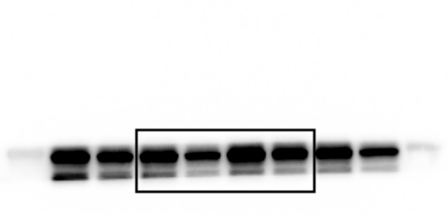

TRIM21

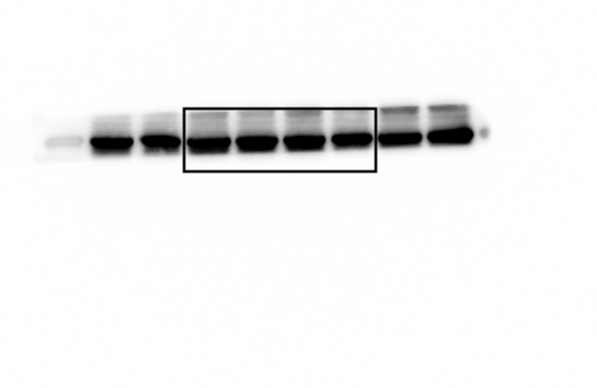

Figure 3

Figure 3H

HeLa-GAPDH

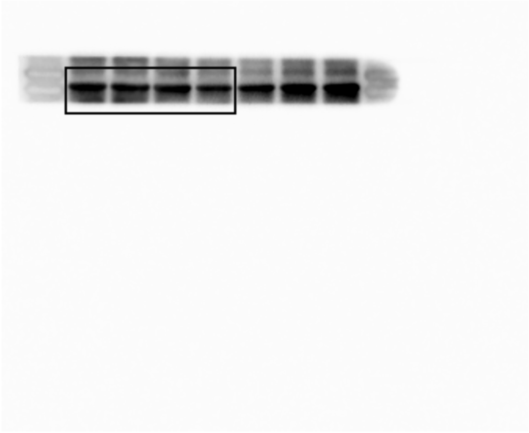

HeLa-NCAPH

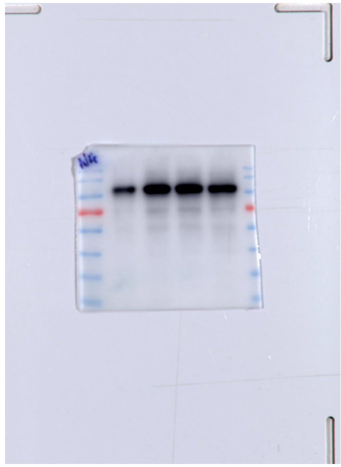

HeLa-TRIM21

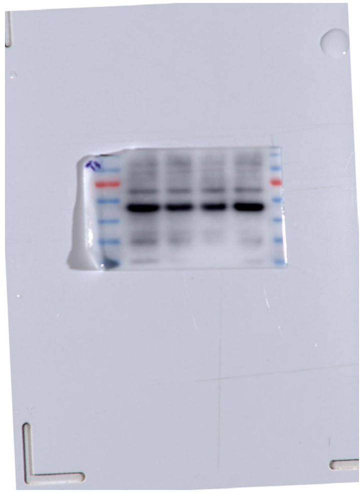

Figure 3I

GAPDH

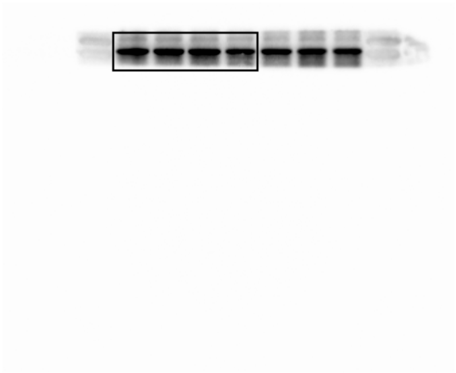

NCAPH

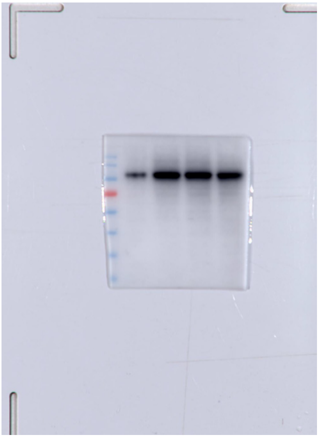

TRIM21

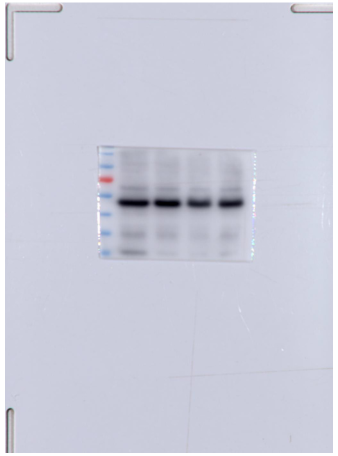

Figure 4

Figure 4B

GAPDH

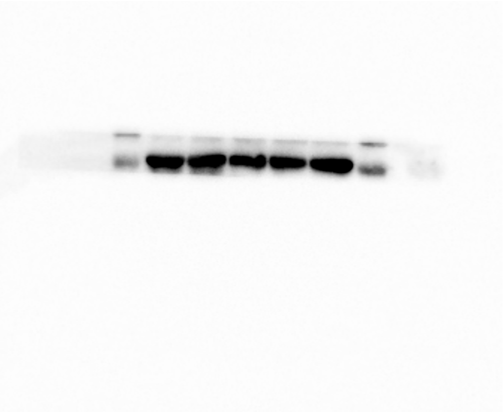

Flag

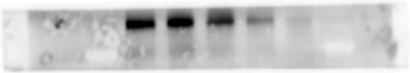

Input-Flag

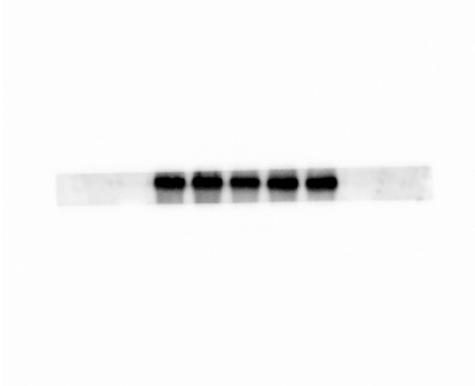

Input-HA

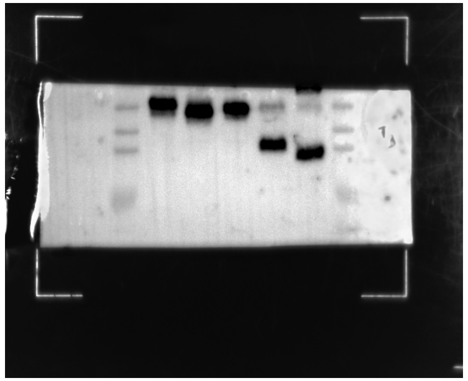

Figure 4C

GAPDH

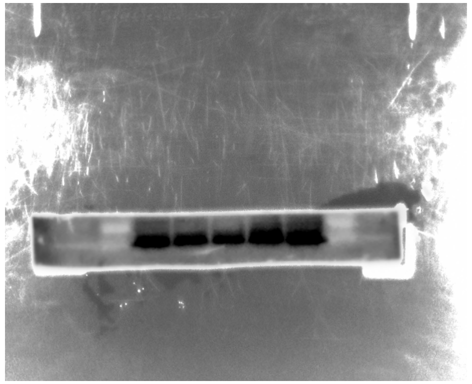

HA

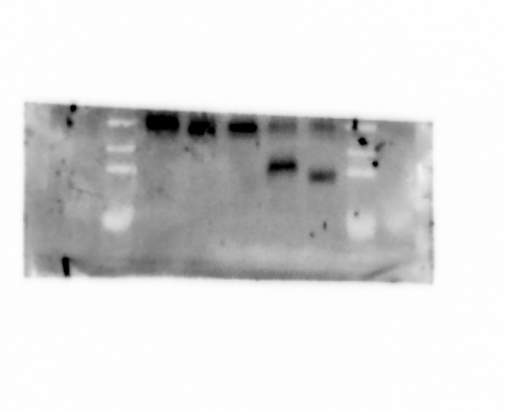

Input-HA

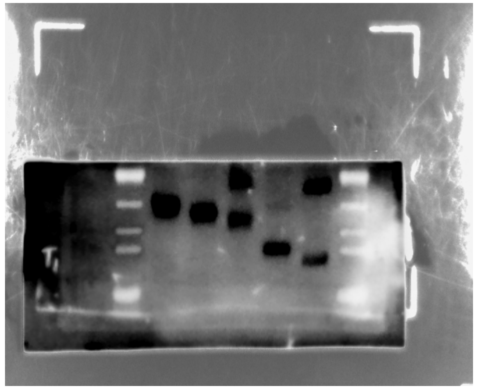

Input-Flag

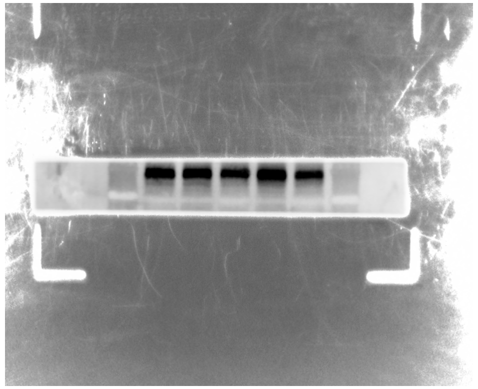

Figure 4

Figure 4D

GAPDH

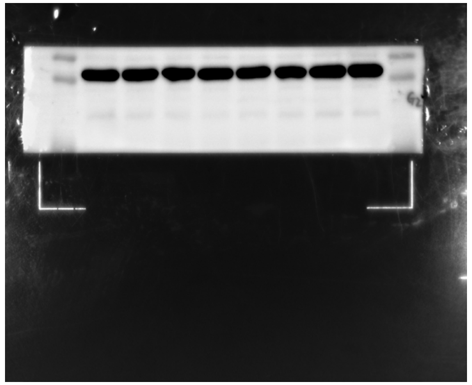

NCAPH

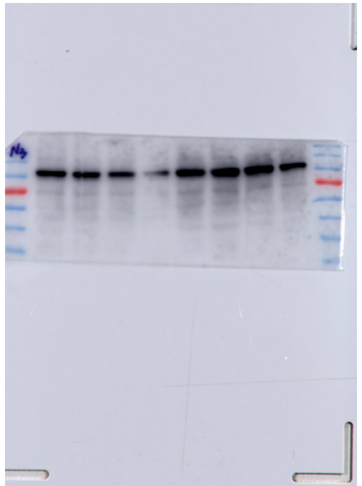

TRIM21

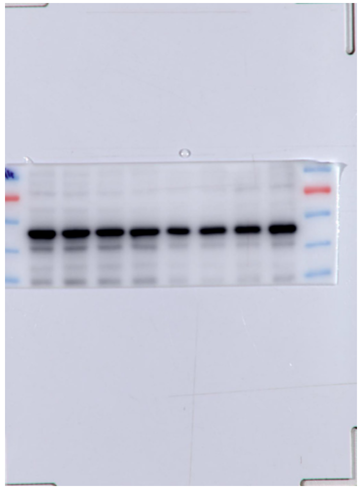

Figure 4E

GAPDH

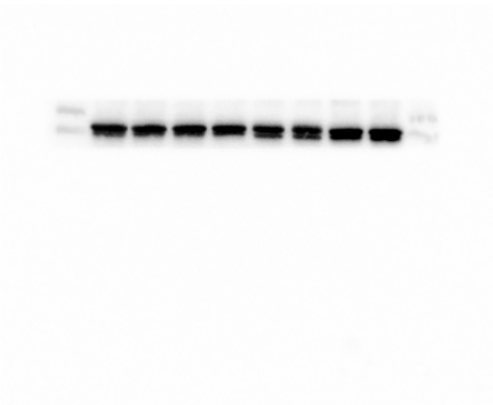

NCAPH

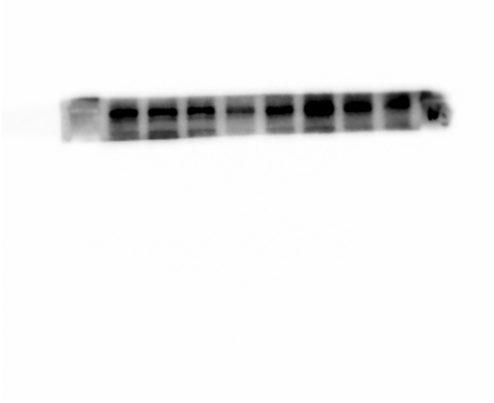

TRIM21

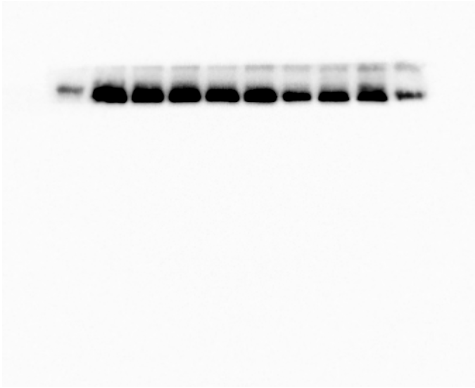

Figure 4

Figure 4H

GAPDH

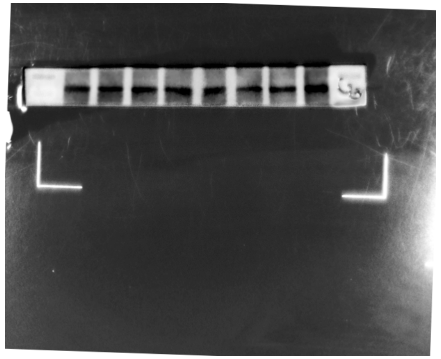

NCAPH

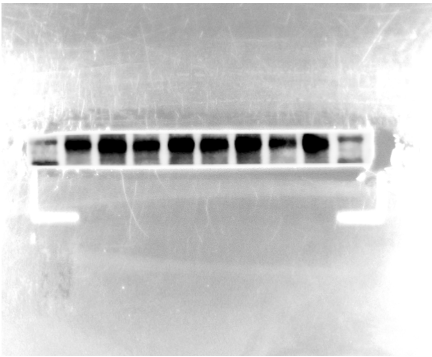

TRIM21

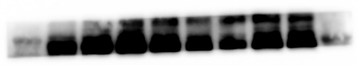

Figure 4L

HeLa+SiHa GAPDH

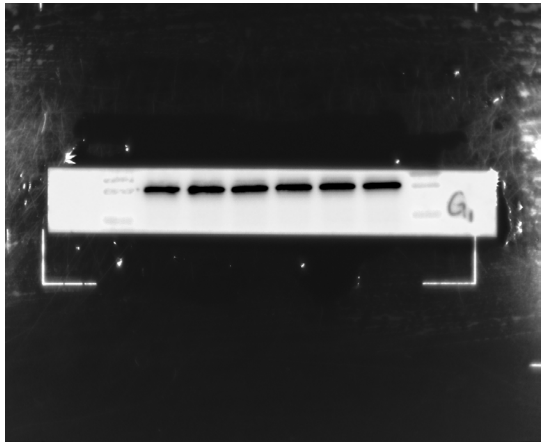

HeLa NCAPH

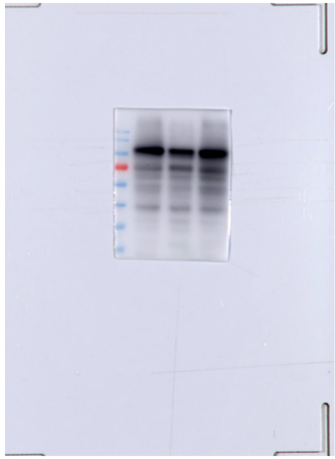

SiHa NCAPH

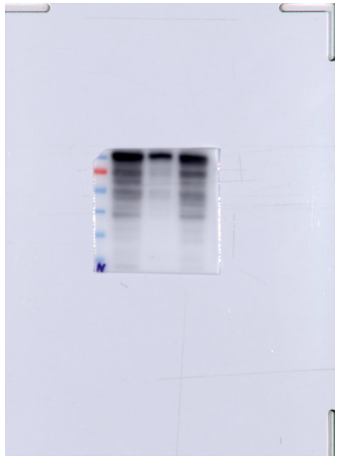

HeLa HA

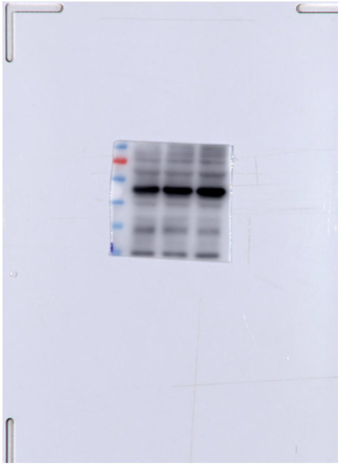

SiHa HA

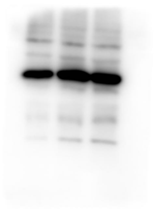

Figure 4

Figure 4I

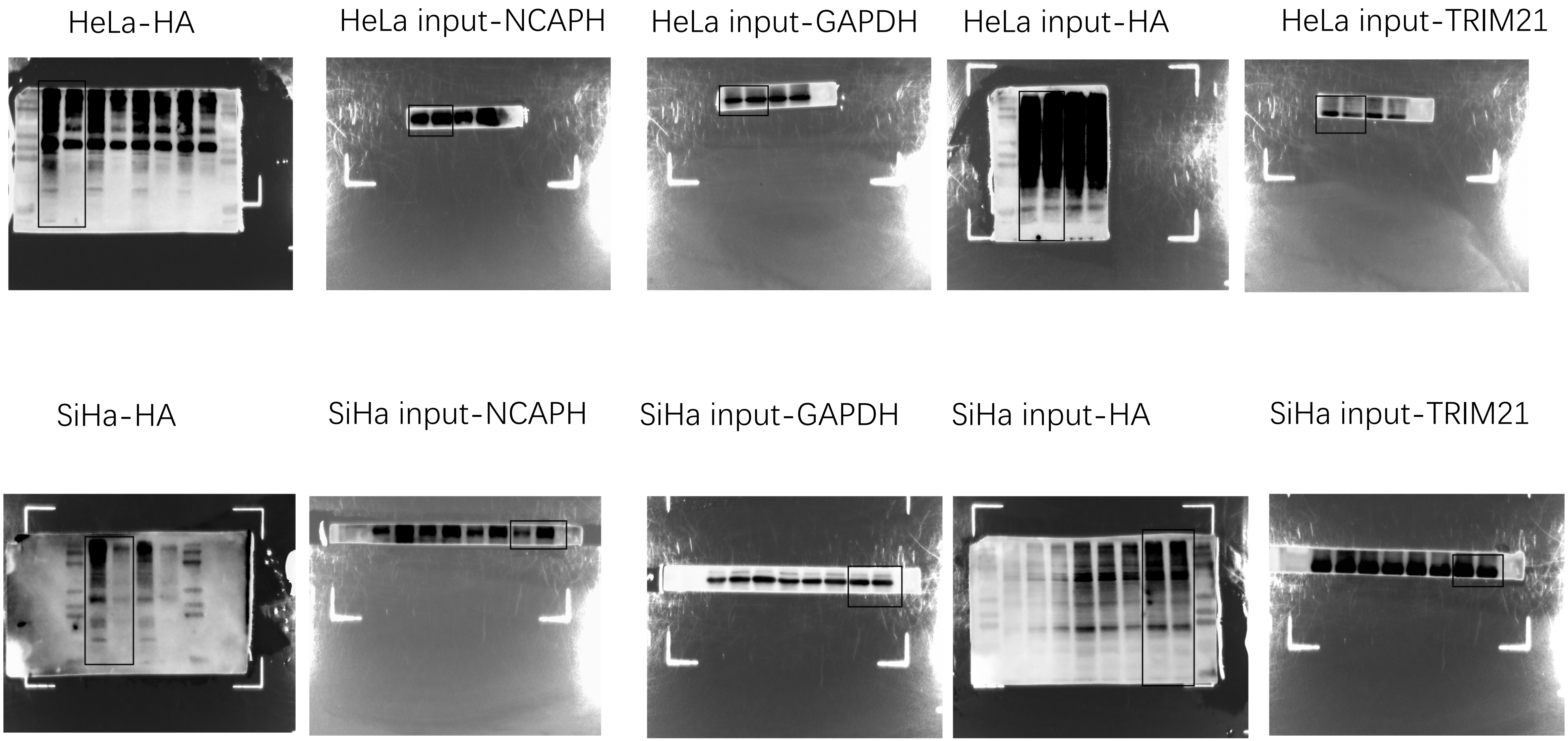

Figure 4

Figure 4J

HeLa-HA

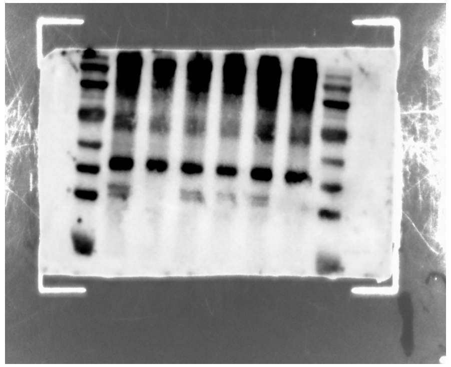

HeLa input-NCAPH

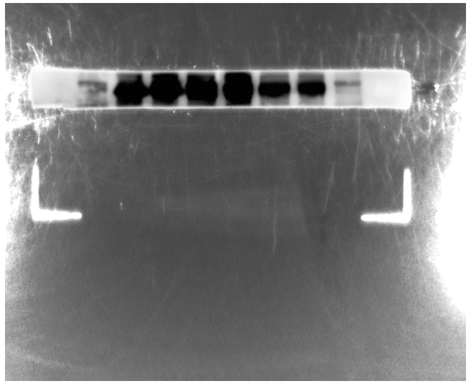

HeLa input-GAPDH

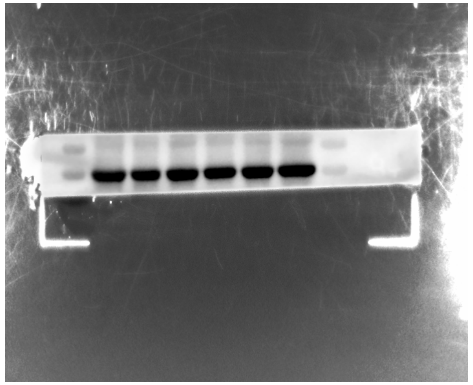

HeLa input-HA

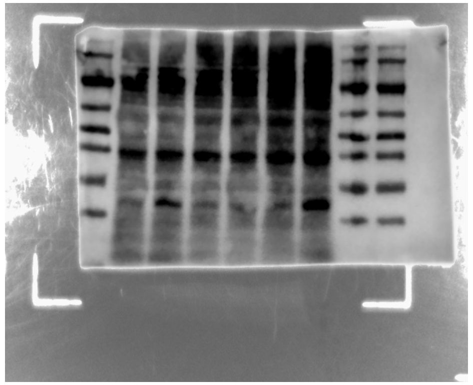

HeLa input-TRIM21

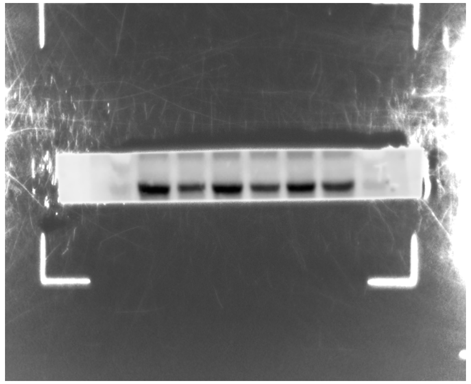

Figure 4K

SiHa-HA

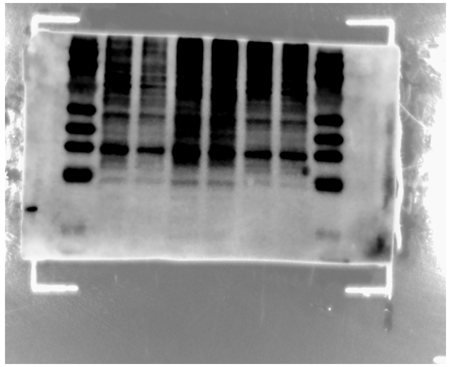

SiHa input-NCAPH

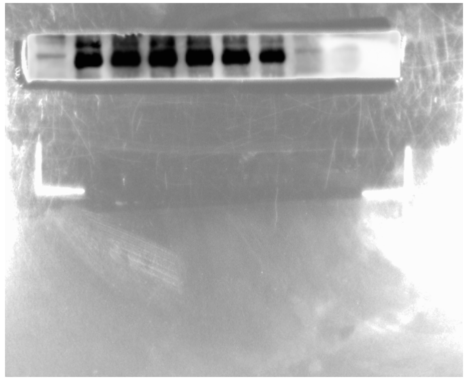

SiHa input-GAPDH

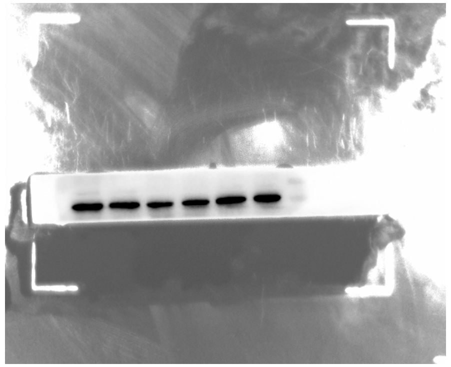

SiHa input-HA

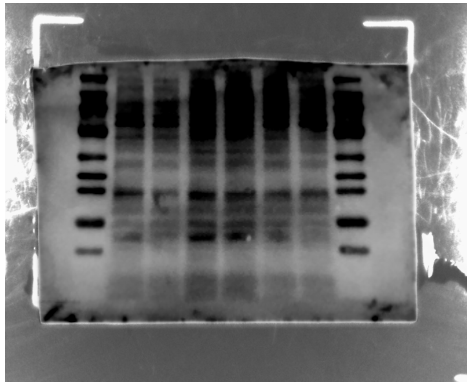

SiHa input-TRIM21

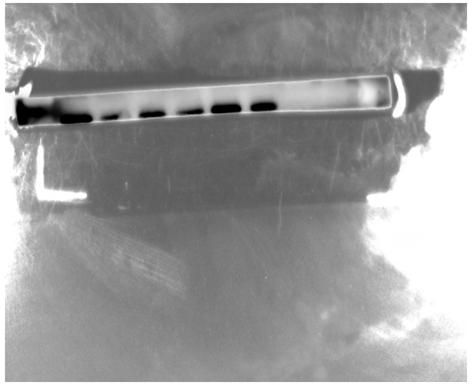

Figure 4

Figure 4M

HeLa-His HeLa input-His HeLa input-NCAPH HeLa input-GAPDH HeLa input-TRIM21

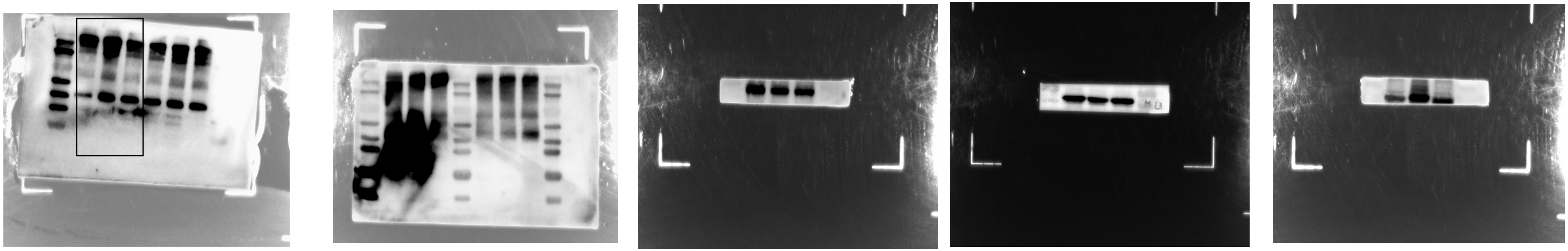

Figure 4N

SiHa-His SiHa input-His SiHa input-NCAPH SiHa input-GAPDH SiHa input-TRIM21

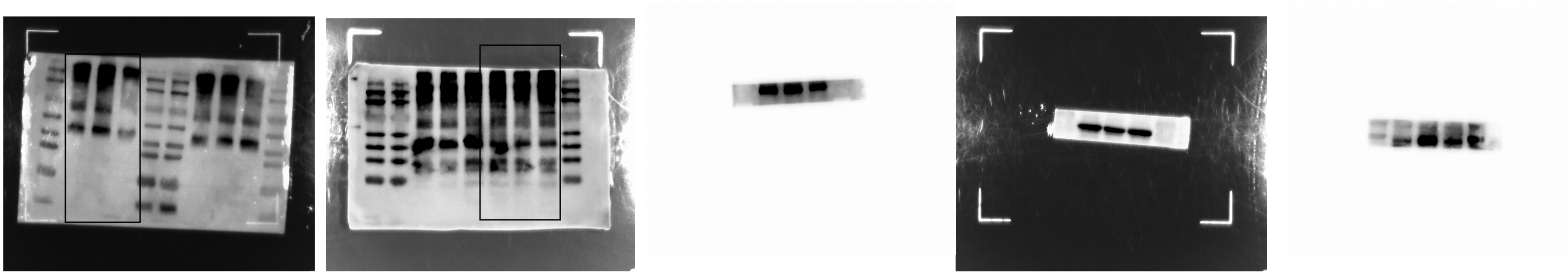

Fig 5B

NCAPH

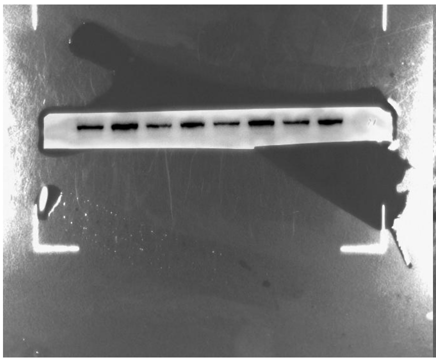

GAPDH

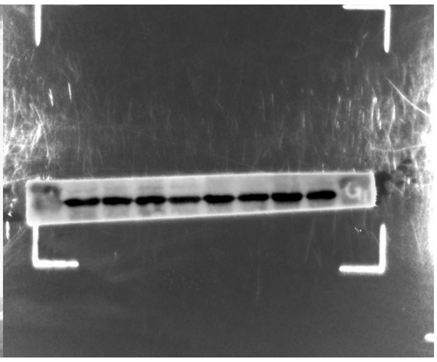

TRIM21

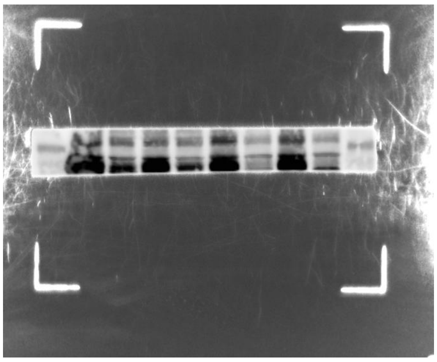

Fig 5F

P62

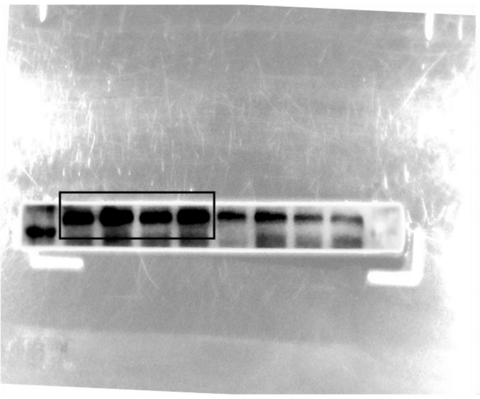

LC3B

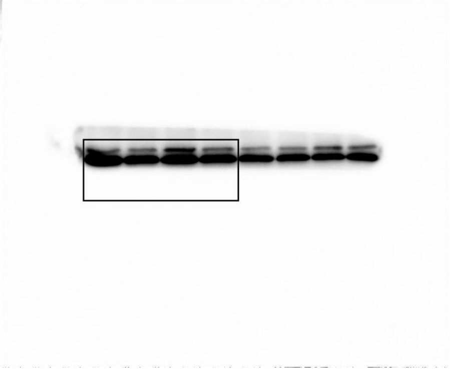

GAPDH

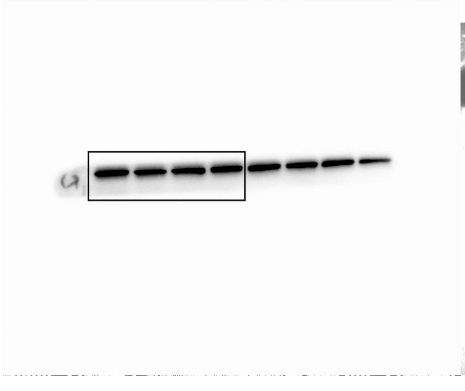

Beclin-1

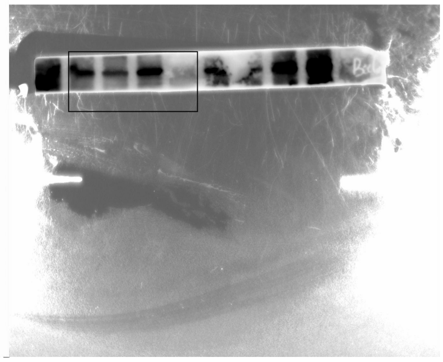

TRIM21

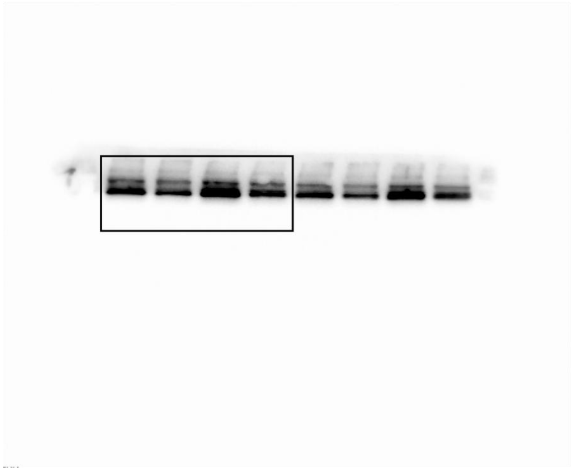

Fig5 M

LC3 B GAPDH

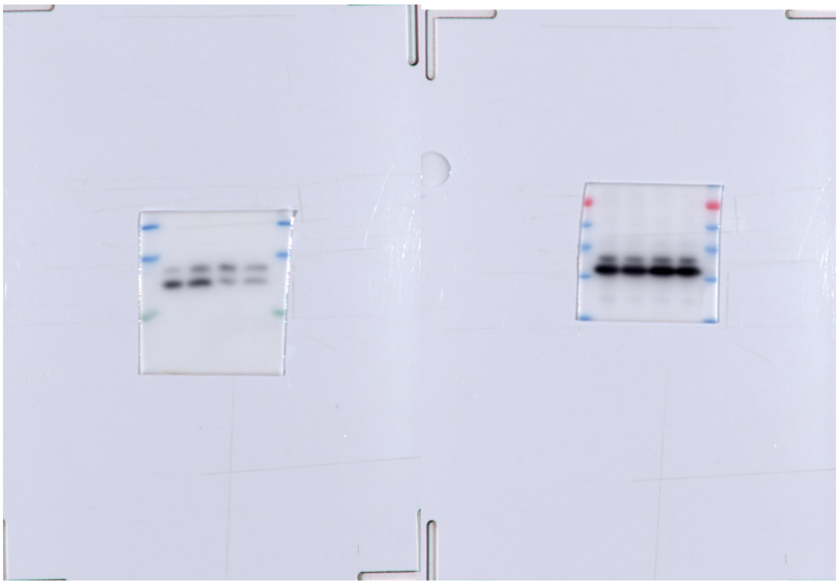

TRIM21 Fig 5N LC3B

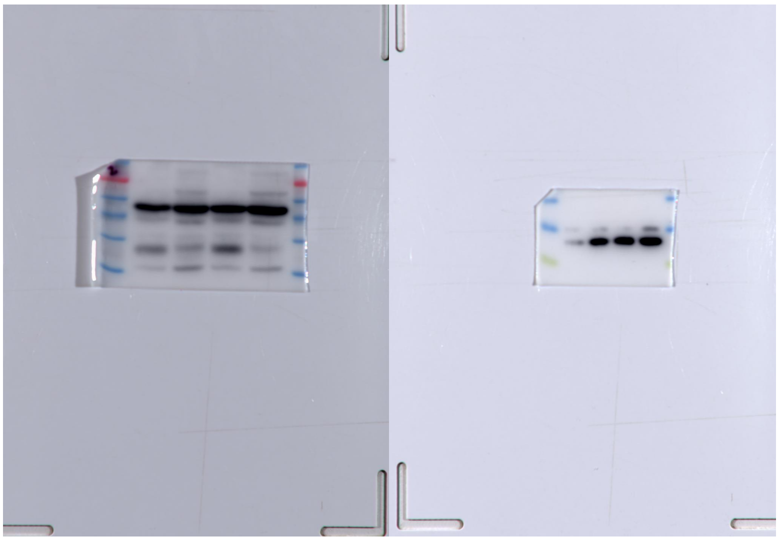

Fig 5N GAPDH

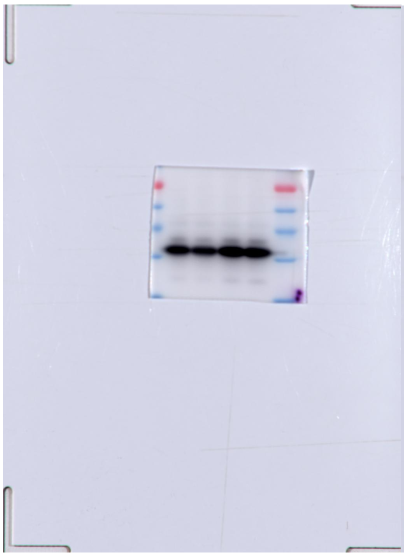

TRIM21

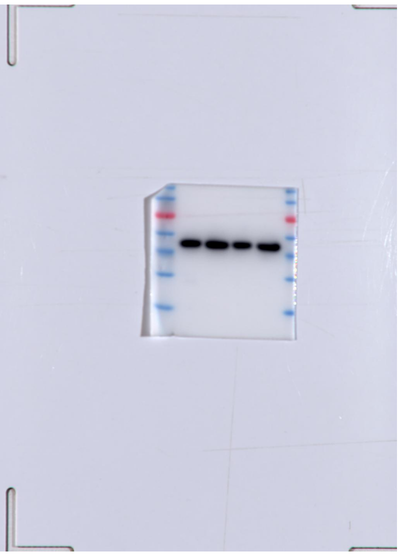

Fig 5 O

LC3B

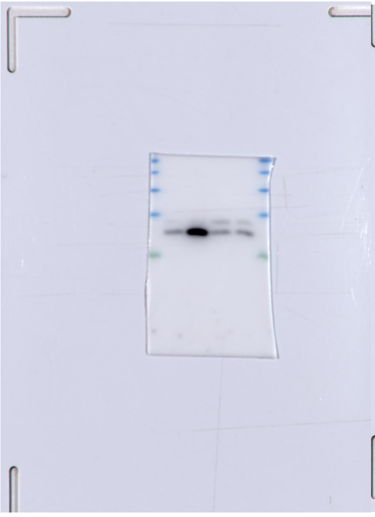

GAPDH

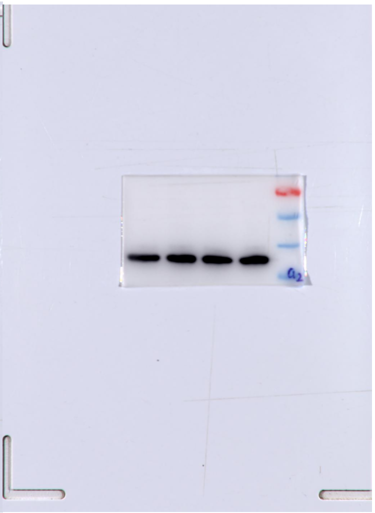

TRIM21

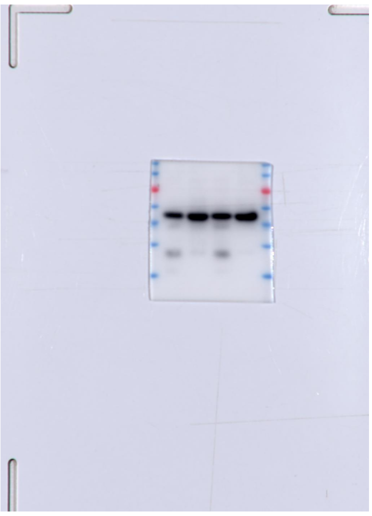

Fig 5P

TRIM21

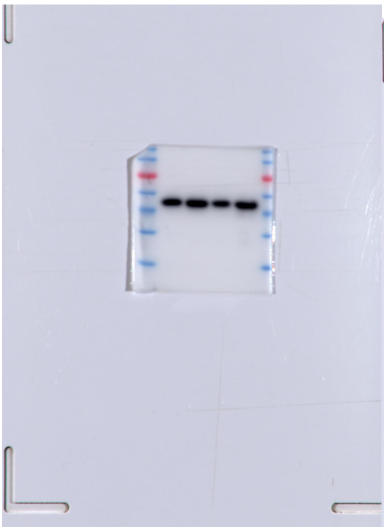

GAPDH

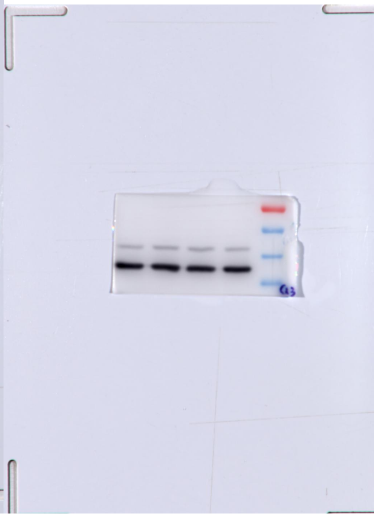

LC3B

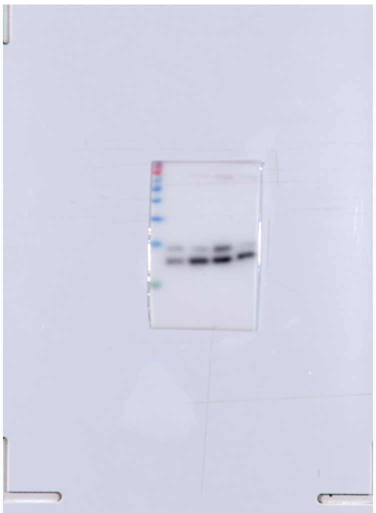

Fig 5Q

GAPDH

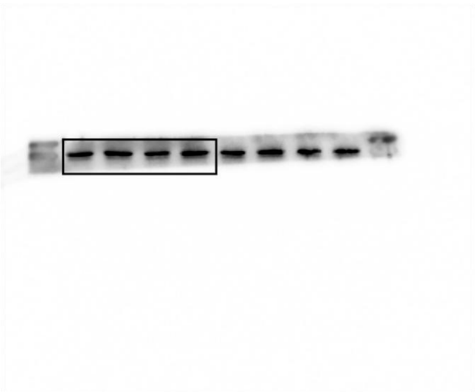

Beclin-1

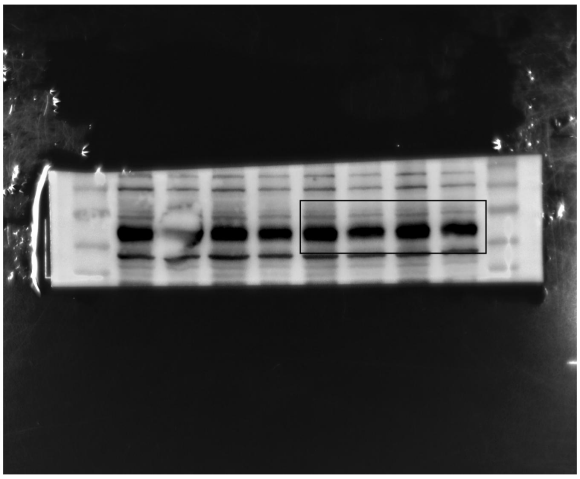

ATG7

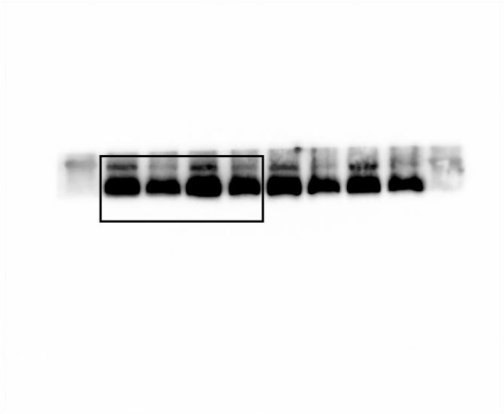

ATG5

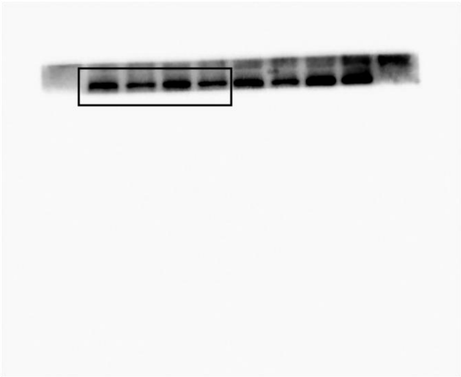

TRIM21

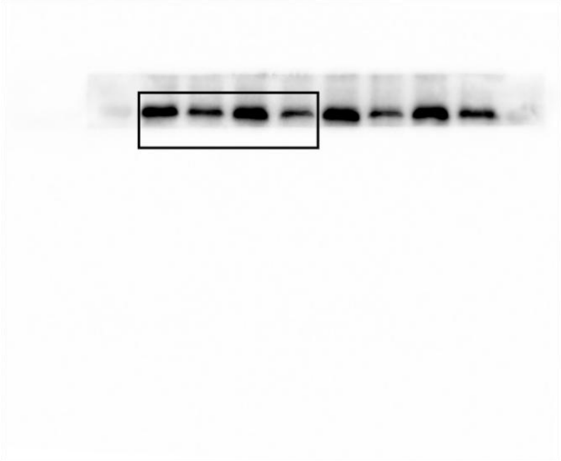

Fig6 I

P62

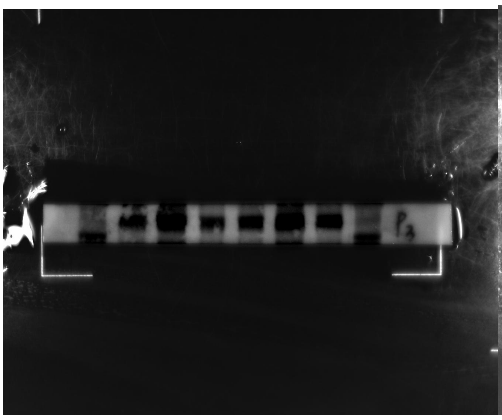

NCAPH

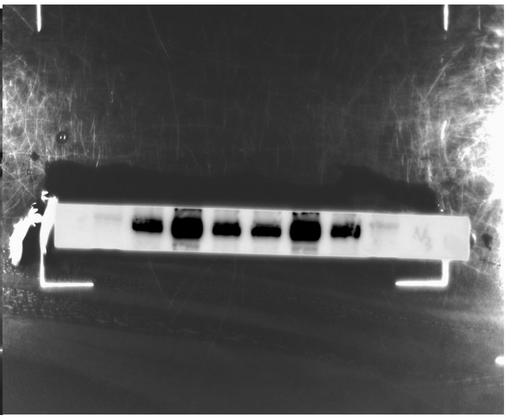

LC3B

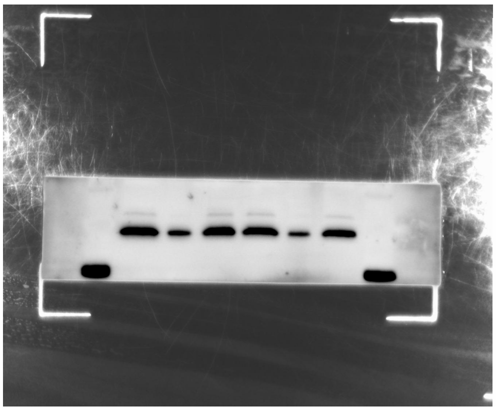

GAPDH

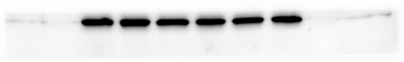

Beclin-1

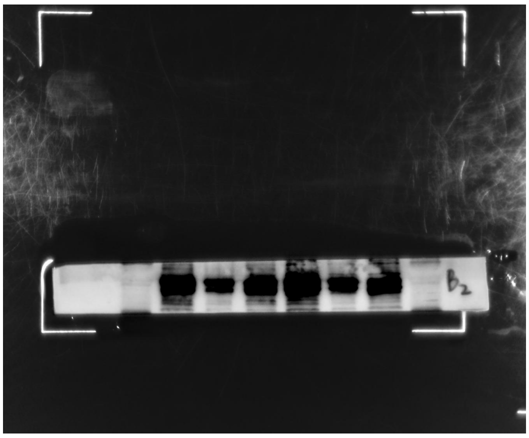

TRIM21

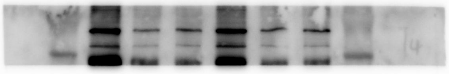

Fig 6 J

LC3 B

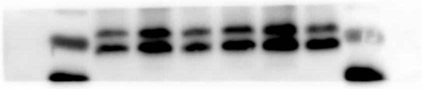

GAPDH

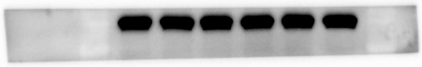

NCAPH

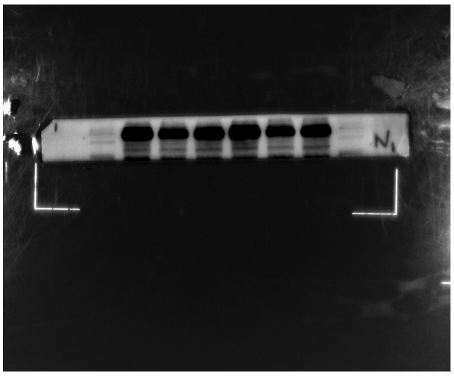

TRIM21

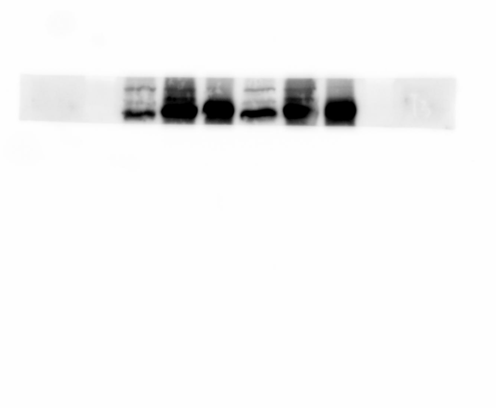

Fig 8E

p-mTOR

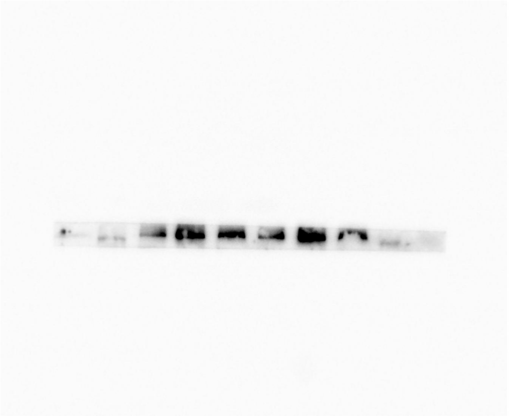

p-AKT

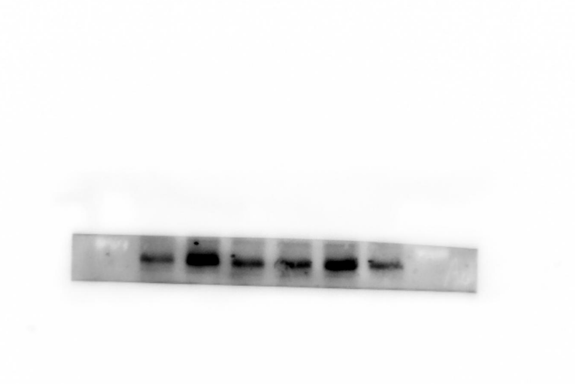

NCAPH

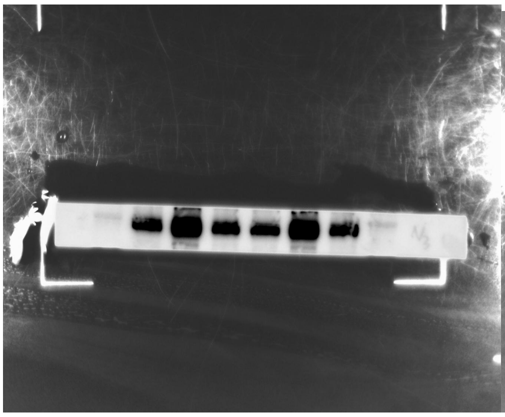

mTOR

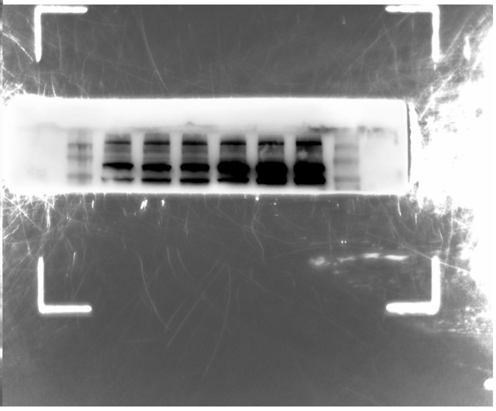

GAPDH

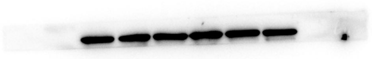

AKT

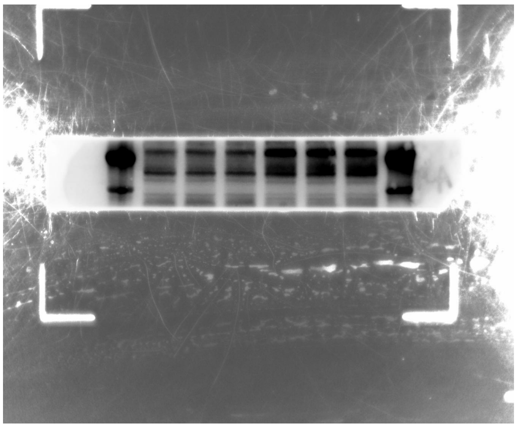

TRIM21

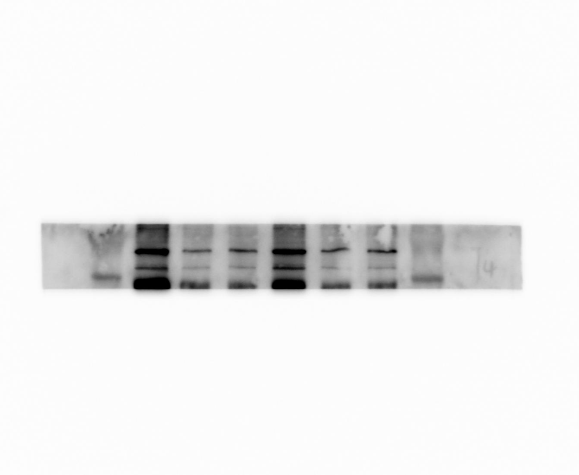

Fig 8 E HeLa

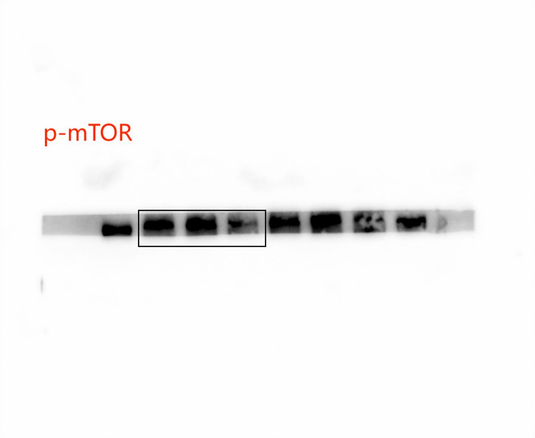

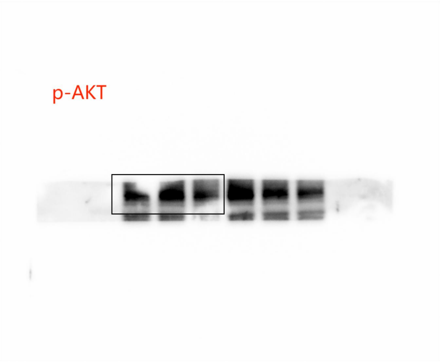

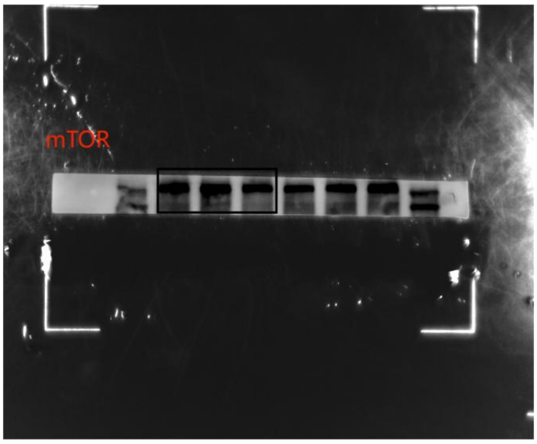

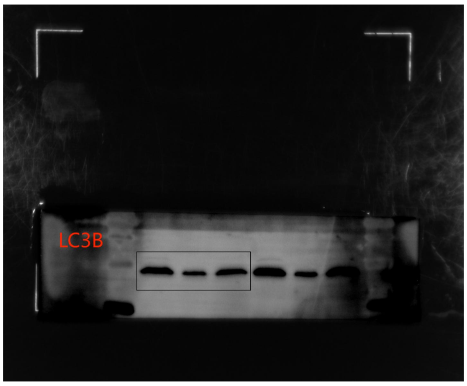

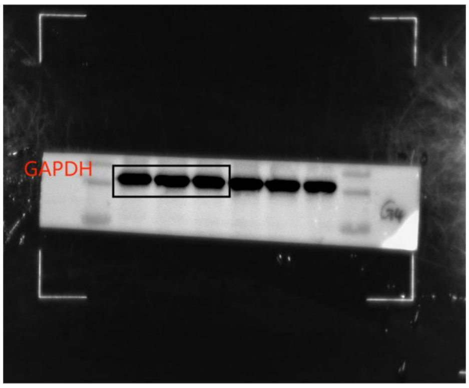

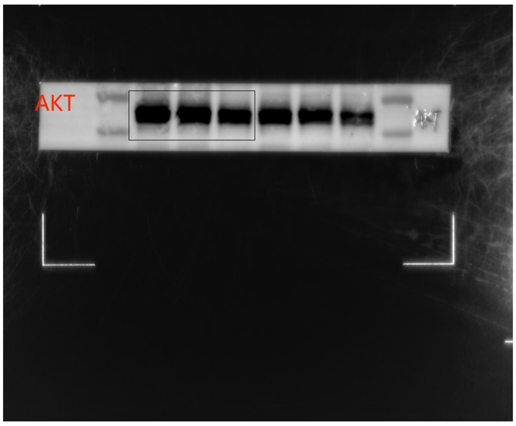

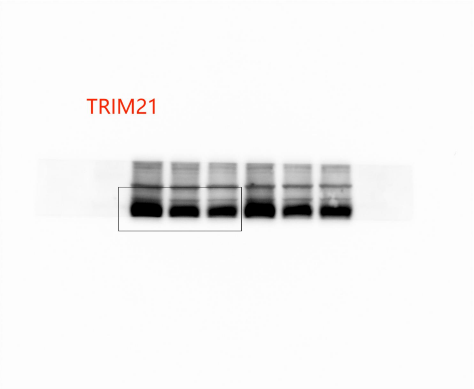

Fig 8 E SiHa

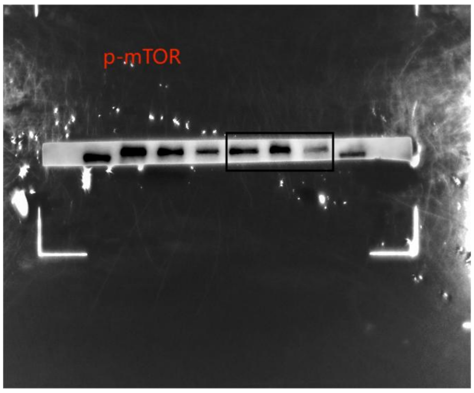

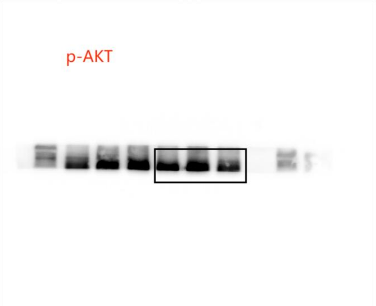

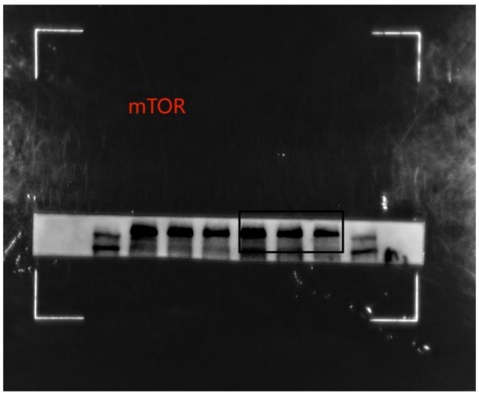
mTOR

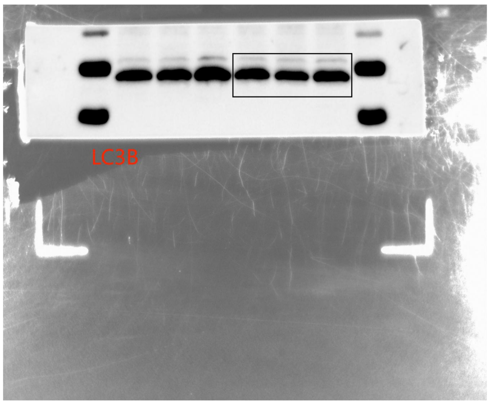
LC3B

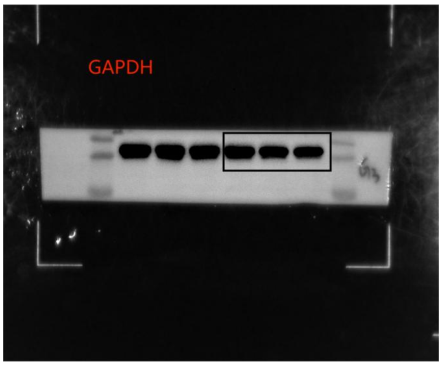
GAPDH

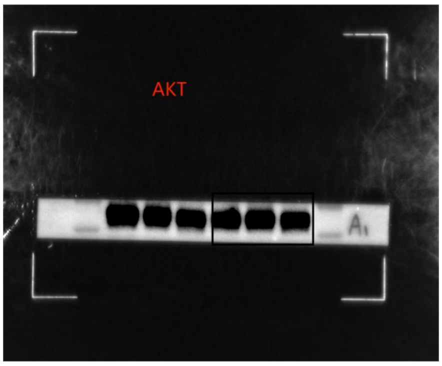
AKT

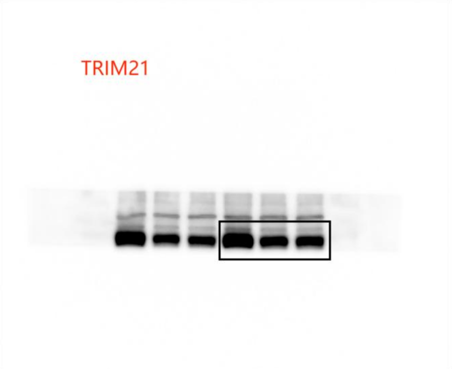
TRIM21
